# RON receptor tyrosine kinase as a critical determinant in promoting tumorigenic behaviors of bladder cancer cells through regulating MMP12 and HIF-2α pathways

**DOI:** 10.1038/s41419-024-07245-w

**Published:** 2024-11-19

**Authors:** Ke-jie Wang, Sha-zhou Ye, Xiao-long Jia, Kai-yun Wang, Xiang-yu Meng, Xin Fei, Shi-jie Ye, Qi Ma

**Affiliations:** 1 Translational Research Laboratory for Urological Diseases, the First Affiliated Hospital of Ningbo University, Ningbo, Zhejiang P. R. China; 2 Comprehensive Genitourinary Cancer Center, the First Affiliated Hospital of Ningbo University, Ningbo, Zhejiang P. R. China; 3 Department of Urology, the First Affiliated Hospital of Ningbo University, Ningbo, Zhejiang P. R. China; 4 Department of Urology, the Affiliated People’s Hospital of Ningbo University, Ningbo, Zhejiang P.R. China; 5 Health Science Center, Ningbo University, Ningbo, Zhejiang P.R. China; 6 Yi-huan Genitourinary Cancer Group, Ningbo, Zhejiang P.R. China

**Keywords:** Metastasis, Bladder cancer

## Abstract

The RON receptor tyrosine kinase is critical in the pathogenesis of various cancer types, however, its role in bladder cancer invasive growth is still largely unknown. Here, we found that over 90% of bladder cancer samples exhibit elevated levels of RON expression, with significantly higher expression levels observed in invasive bladder cancer compared to non-invasive bladder cancer. In vitro, RON activation resulted in increased bladder cancer cell migration and invasiveness. Results from mRNA sequencing and transcriptome analysis further demonstrated that MMP12, a downstream molecule of RON, is functionally involved in regulating RON-mediated bladder cancer cell migration and invasiveness. The underlying mechanism appeared to be the RON-mediated inhibition of HIF-2α ubiquitination, which is channeled through the activation of the JNK signaling pathway. Consequently, the activated JNK pathway increased MMP12 expression, ultimately driving bladder cancer cell migration and invasion. As evident in bioinformatics and dual-luciferase reporter assays, the RON mRNA at its 3′-untranslated regions specifically interacted with hsa-miR-659-3p. The binding of hsa-miR-659-3p downregulated the RON gene expression, attenuating the receptor-mediated tumorigenic activities of bladder cancer cells in vitro and in vivo. In conclusion, aberrant RON expression in bladder cancer cells and MMP12 and HIF-2α activities form a functional axis that causes increased bladder cancer cell migration and invasion. The fact that hsa-miR-659-3p downregulates RON expression indicates its critical role in attenuating RON-mediated tumorigenic effect on bladder cancer cells. These findings highlight the importance of RON targeting as a therapeutic means for potential bladder cancer therapy.

## Introduction

Invasive growth of bladder cancer cells into the neighboring tissue is a critical event directly associated with tumor malignant progression, chemoresistance, and clinical outcome [1]. Pathologically, tissue invasion by bladder cancer cells, according to their depths of penetration, is categorized into two groups, including one that invades into the bladder muscle layers (muscle-invasive bladder cancer, MIBC) and another without the muscle penetration (non-muscle invasive bladder cancer, NMIBC) [2]. Endoscopic resection in combination with intra-vesicular chemotherapy or the local instillation of bacillus Calmette–Guerin is commonly used to treat NMIBC [3]. In contrast, patients with MIBC must undergo a radical cystectomy to remove the invading cancerous lesions [4]. Currently, antibody–drug conjugates (ADCs) and immune checkpoint inhibitors (ICIs) have been applied for advanced bladder cancer, resulting in reduced disease recurrence and delayed tumor progression [5]. Nevertheless, clinical observations indicate that MIBCs behave more malignantly than NMIBCs and are likely to progress rapidly with poor clinical prognosis, which ultimately leads to patient death [2]. Considering these facts, it is essential to understand the cellular mechanisms underlying malignant behaviors of advanced bladder cancer cells, including their migration and invasiveness capabilities. Outcomes from these studies should help us not only understand the biology of bladder cancer pathogenesis but also facilitate the identification of novel therapeutic targets for the development of effective therapeutics to combat this malignant disease.

The receptor tyrosine kinase (RTK) receptour d’Origine nantais (RON), also known as macrophage-stimulating protein (MSP) 1 receptor, belongs to the unique MET RTK family, which contributes significantly to epithelial tumorigenesis and chemoresistance [6]. Activation of RON, either by ligand MSP stimulation or by aberrant expression, stimulates mitogen-activated protein kinase (MAPK), phosphatidylinositol 3 kinase (PI3K), and other signal pathways, resulting in increased cancerous cell proliferation, survival, migration, and matrix invasion [7]. Clinically, aberrant RON expression and signaling have been shown to have prognostic values for several types of cancers, including those from the breast, colon, and pancreas [8, 9]. Recently, some studies have also found that altered RON expression in bladder cancer is positively associated with tumor histology grading, larger tumor size, non-papillary contour, and higher tumor staging [10]. Our previous study has also demonstrated that RON is overexpressed with more than 50% positivity in primary bladder cancer samples. The frequencies of the cancerous RON positivity are significantly higher than that from normal or tumor-surrounding tissues [11]. More importantly, increased RON expression is closely related to bladder cancer cell invasion into the local tissues and their distant metastasis [12]. These findings suggest that aberrant RON expression and activation are the pathogenic factors contributing significantly to bladder cancer malignancy. Understanding cellular mechanisms by which RON regulates bladder cell invasive growth has clinical relevance and is urgently needed.

Studies presented in this communication are our attempt to determine the cellular mechanism(s) by which aberrant RON signaling facilitates bladder cancer cell migration and invasiveness. Specifically, we focus our attention on the RON-mediated activation of 2 effector molecules, including matrix metalloproteinase-12 (MMP12) and hypoxia-inducible factor-2α (HIF-2α), both of which are known to play an important role in regulating cancerous cell migration and tissue penetration [13, 14]. Moreover, we studied the role of certain microRNA species (miRNAs) as endogenous regulators in modulating RON expression and its associated tumorigenic activities in bladder cancer cells. Accumulated evidence has demonstrated that certain miRNAs can regulate the cell surface receptor expression by downregulating the target molecule mRNA expression [15]. Currently, the role of miRNAs in controlling RON expression has not been studied. Through a series of experiments, we obtained results demonstrating that aberrant RON expression and signaling are critical in regulating bladder cancer cell malignant activities. MMP12 and HIF-2α are the downstream effector molecules of RON, which are highly involved in RON-mediated bladder cancer cell migration and invasiveness. Furthermore, we discovered that miRNA hsa-miR-659-3p specifically interacts with the RON mRNA, resulting in its degradation, and affecting the RON-mediated tumorigenic activity. These findings open an avenue for using targeted therapeutics to specifically inhibit RON-mediated bladder cancer cell malignancy for therapeutic intervention in the future.

## Results

### Aberrant RON expression in association in vitro with increased bladder cancer cell migration and matrix invasion

**Fig. 1:**
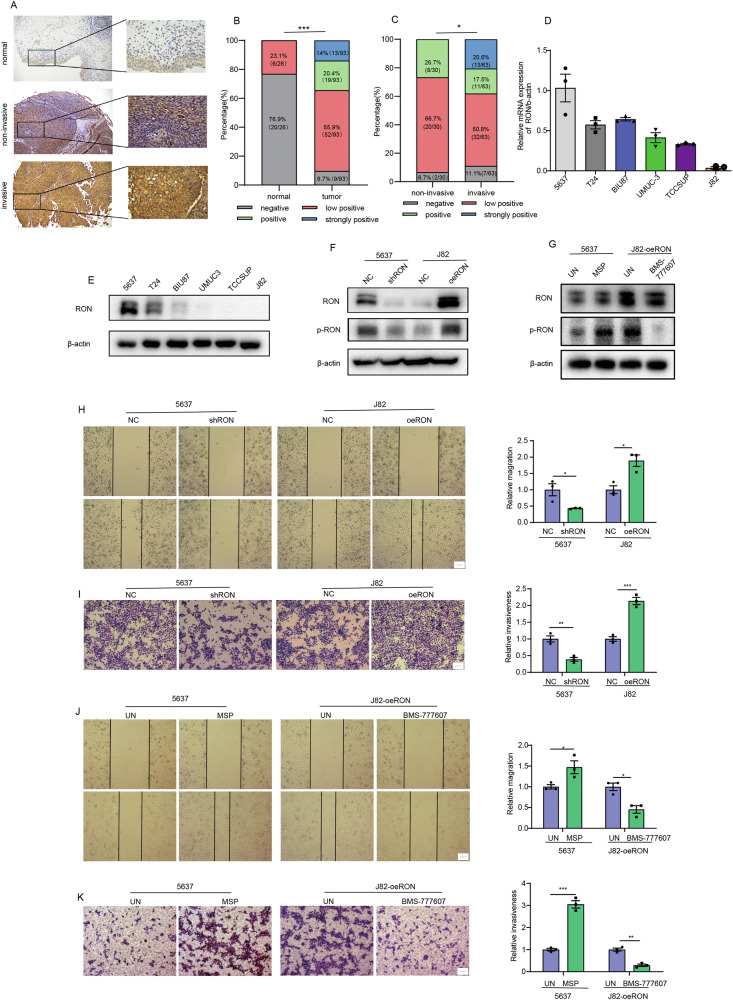
Anomalous RON expression correlates with heightened migration and matrix invasion of bladder cancer cells. **A** Immunohistochemical staining was performed to assess RON protein expression in bladder cancer (*n* = 93) and adjacent normal tissues (*n* = 26). **B** The IHC analysis revealed distinct staining patterns of RON protein expression in bladder cancer and adjacent normal tissues, with a total of 119 samples analyzed, including 26 normal tissues and 93 bladder cancer tissues. **C** The IHC analysis demonstrated varying staining patterns of RON protein expression in invasive and non-invasive bladder cancer tissues, with a total of 93 samples analyzed, including 30 non-invasive and 63 invasive bladder cancer tissues. **D** The relative mRNA expression level of RON in bladder cancer cell lines was assessed using quantitative real-time polymerase chain reaction (qRT-PCR, with 5637 cells serving as the control). **E** The protein expression level of RON in bladder cancer cell lines was determined through western blot analysis. **F** A total of 5637 cells were subjected to transfection with either the shRON Lentiviral vector or the corresponding empty lentiviral vector to generate stable cell lines with low RON expression levels (designated as 5637-NC and 5637-shRON, respectively). Similarly, J82 cells were transfected with the RON Lentiviral vector or the corresponding empty lentiviral vector to establish stable cell lines with overexpressed RON (designated as J82-NC and J82-oeRON, respectively). The expression of RON and p-RON were subsequently validated using Western blot analysis. **H** The migratory capacity of 5637-NC, 5637-shRON, J82-NC, and J82-oeRON cells was assessed using a wound healing assay. Representative images were obtained at a magnification of 40× (Scale bar 1 µm). **I** The invasion ability of 5637-NC, 5637-shRON, T24-NC, and T24-oeRON cells was evaluated by trans-well assay. Representative images were captured at a magnification of 40× (Scale bar 1 µm). In total, 5637 cells were stimulated with MSP for 24 h, and J82-oeRON cells were treated with RON inhibitor BMS-777607 for 24 h. **G** Western blot analysis was performed to assess the phosphorylation status of RON. **J** Cell migration was assessed using a wound-healing assay. Representative images were obtained at a magnification of 40× (Scale bar 1 µm). **K** The invasion ability of the cells was evaluated by trans-well assay. Representative images were captured at a magnification of 40× (Scale bar 1 µm). Experiments in **D**–**K** were repeated three times (*n* = 3).

To explore the role of RON in bladder cancer, we used immunohistochemical staining to detect RON expression in 93 bladder cancer tissues and 26 normal bladder tissues. The results showed that the positive expression rate of RON in bladder cancer tissues was 90.3%, while that in normal tissues was only 23.1% (Fig. 1A, B). Moreover, it is noteworthy that the level of RON expression was markedly elevated in cases of invasive bladder cancer compared to non-invasive bladder cancer (Fig. 1A, C). To study the role of RON in bladder cell tumorigenesis, a panel of 6 bladder cancer cell lines (5637, T24, BIU87, UMUC-3, TCCSUP, and J82) were analyzed for the RON expression using qRT-PCR and Western blot methods (Fig. 1D, E). At protein levels, cell line 5637 showed the highest RON expression. Moderate levels of RON were observed in cell line T24. BIU87, UMUC3, TCCSUP, and J82 cells only expressed a trace amount of RON (Fig. 1E). Stable cell lines were then generated either with RON knockdown (5637-shRON) or RON overexpression (J82-oeRON), together with their corresponding control cell lines including 5636-NC and J82-NC (Fig. 1F). These cell lines were tested in the trans-well and wound healing assays to determine their migrating and invasive activities. Results in Fig. 1H showed that 5637-NC cells display an increased migrating activity, which was significantly attenuated upon the silencing of RON. However, J82 cells showed increased cell migration activity following the overexpression of RON. Similar results were also documented in the cell-matrix invasion assay, in which the knockdown of the RON expression dramatically decreases the 5637 cell-mediated matrix invasiveness. Consistently with these findings, we observed that increased RON expression by J82 cells results in enhanced invasive activities in vitro (Fig. 1I). To confirm aberrant RON expression leading to constitutive activation in the absence of ligand stimulation [16], we used Western blot methods to study the RON phosphorylation status. Results in Fig. 1F showed the diminished RON phosphorylation in 5637-shRON cells and the increased RON phosphorylation in J82-oeRON cells. Stimulation of 5637 cells showing increased RON expression with MSP appeared to further enhance RON phosphorylation (Fig. 1G), which is associated with increased cell migration and invasive activity (Fig. 1J, K). Moreover, treatment of J82-oeRON cells with RON-specific inhibitor BMS-777607 significantly inhibited RON phosphorylation levels (Fig. 1G), This effect was also associated with diminished cell migration and invasive activity (Fig. 1J, K). Taken together, results in Fig. 1 demonstrate that increased RON expression and activation promote cell migration and matrix invasion of bladder cancer.

### Identification of MMP12 by cellular expressional profiling as a key protein associated with increased RON expression in bladder cancer cells

**Fig. 2:**
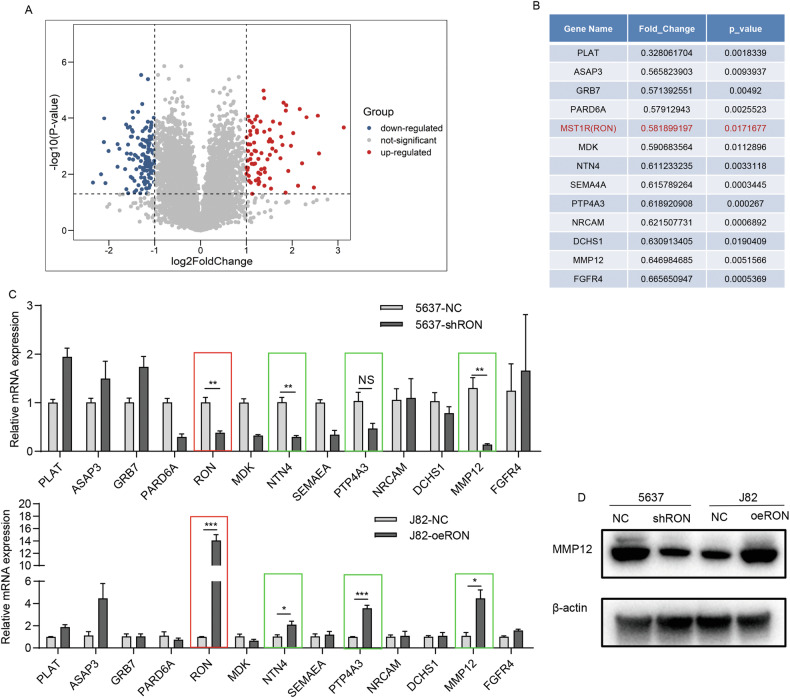
Identification of MMP12 through cellular expressional profiling as a pivotal protein linked to increased RON expression in bladder cancer cells. **A** Significant alterations in mRNA expression were observed in 5637-shRON cells compared to 5637-NC cells, as determined by RNA-seq analysis. **B** Through KEGG and GO analysis, as well as combining annotations in the GENE database, 13 candidate genes closely associated with migration and invasion were identified among genes positively correlated with RON expression. **C** Validation of candidate genes positively correlated with RON expression in 5637-shRON, 5637-NC, J82-oeRON, and J82-NC groups was conducted using qRT-PCR. **D** The protein expression levels of MMP12 were assessed in 5637-shRON, 5637-shNC, J82-oeRON, and J82-oeNC cells by western blot. Experiments in **C**, **D** were repeated three times (*n* = 3).

To understand the role of RON in malignant behaviors of bladder cancer cells in more detail, we applied the cellular mRNA expression profiling method to 5637-shRON and 5637-NC cells to determine any expressional differences between this pair of cell lines. Results from the mRNA sequencing showed a total of 215 genes differentially expressed, of which 80 genes were up-regulated and 135 downregulated (Fig. 2A). To find genes that are directly regulated by RON, we first focused on the 135 down-regulated genes. We performed KEGG and GO analysis on these genes and combined the annotations in the GENE database to screen 13 genes that are directly related to cell migration and invasion (which include RON) (Fig. 2B**)**. We then used qRT-PCR to confirm the expression of these 13 genes in 5637-shRON and 5636-NC paired and in J82-oeRON and J82-NC paired cells. Results in Fig. 2C showed that the mRNA expression levels of NTN4, PTP4A3, and MMP12 were positively correlated with the RON expression in bladder cancer cells. Among these 3 genes analyzed, changes in the MMP12 gene expression are mostly associated with the changes in the RON gene expression. This observation was further confirmed by Western blot analysis using the 2 cell pairs as described above. As shown in Fig. 2D, levels of the MMP12 expression in both 5637-shRON and 5636-NC paired, and J82-oeRON and J82-NC paired cells are positively correlated with the level of the RON expression.

### MMP12 as an effector molecule in RON-mediated bladder cancer cell migration and matrix invasion

**Fig. 3:**
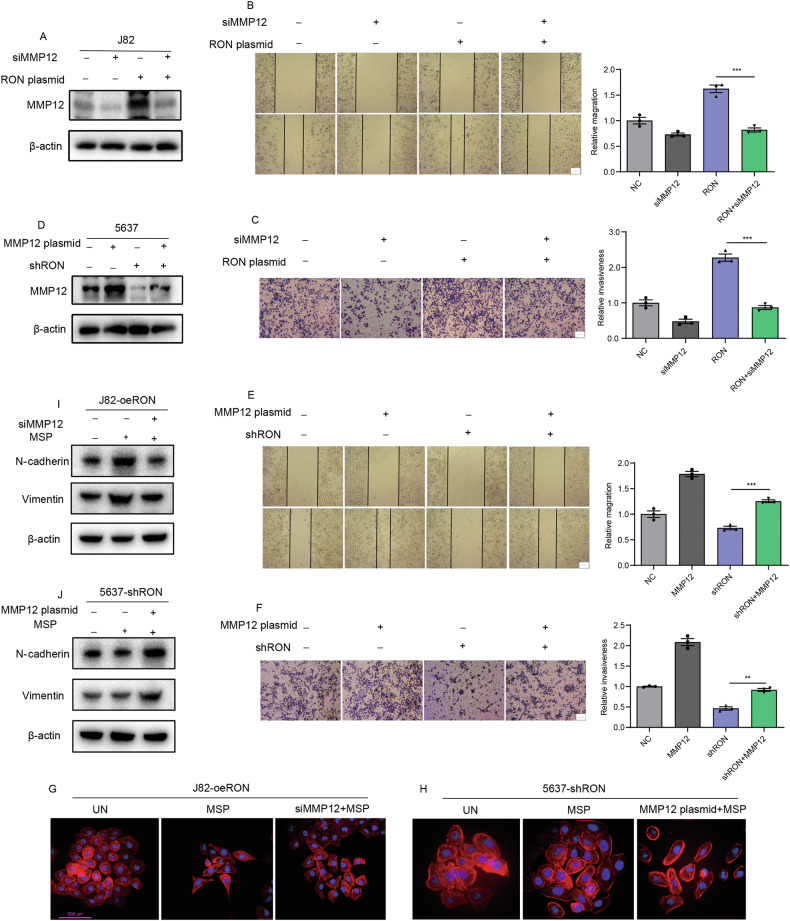
MMP12 functions as an effector molecule in the process of RON-mediated bladder cancer cell migration and matrix invasion. **A** J82 cells were transduced with siRNA targeting MMP12 and an overexpression plasmid for RON. Western blot analysis was utilized to confirm the expression of MMP12. **B** The migratory capacity was assessed through a wound-healing assay. **C** The invasive potential was evaluated using a trans-well assay. **D** In total, 5637 cells were transduced with an overexpression plasmid for MMP12 and a shRON plasmid. Western blot analysis was employed to validate the expression of MMP12. **E** The migratory ability was assessed through a wound-healing assay. **F** The invasion ability was evaluated by trans-well assay. The scale bar above is all 1 µm. Actin-Tracker Red-555 and DAPI were utilized for staining the cytoskeleton and nucleus, respectively, with representative images displayed in Fig. (**G**) and (**H**). The scale bar measures 500 µm. **I**, **J** Western blot analysis was used to confirm the expression of N-cadherin and vimentin. All experiments above were repeated three times (*n* = 3).

Identification of MMP12 prompted us to study whether RON-mediated bladder cancer cell invasive growth is channeled through this functional protein. To this end, J82 cells transfected with the specific siRNA for MMP12 silencing or with the RON cDNA for receptor overexpression were generated. Also, 5637 cells engineered to overexpress MMP12 or its knockdown by the specific shRNA were established, as evidenced by the Western blot analysis (Fig. 3A, D). Results from Fig. 3B, C showed that increased RON expression in bladder cancer cells causes enhanced cellular migration and invasion. This effect was significantly diminished in cells showing MMP12 knockdown. Consistent with a previous report in other tumors [17, 18], increased MMP12 expression alone was also sufficient to cause bladder cancer cell invasion and migration (Fig. 3E, F). Furthermore, our findings indicate that MSP activation of RON in 5637 cells leads to an upregulation of MMP12 expression (Fig. S1A), thereby enhancing cell migration and invasion activity. This effect was attenuated by knocking down MMP12 (Fig. S1C, D). Conversely, inhibition by BMS-777607 of RON activity in J82-oeRON cells results in a downregulation of MMP12 expression (Fig. S1B), which was associated with diminished cell migration and invasion. We further observed that the effect of RON inhibition can be reversed through MMP12 overexpression (Fig. S1E, F). Another interesting finding from our studies is the effect of RON expression and activation in regulating cell morphological changes of bladder cancer cells, known as epithelial to mesenchymal transition (EMT) [19]. As shown in Fig. 3G, overexpression of RON in J82-oeRON cells resulted in visible cell morphological changes. The addition of MSP further altered cell morphologies from spindle to elongated and polygonal shapes, which appear to have the characteristics of mesenchymal cells. To our surprise, the RON-mediated EMT-like appearance in bladder cancer cells was prevented to a certain extent when the MMP12 expression was knocked down by the specific siRNA (Fig. 3G). To verify this result, we treated 5637-shRON cells with MSP to see any morphological changes. As expected, we did not observe significant changes in cell morphologies in these cells with the diminished RON expression. Again, the MMP12 overexpression in these cells led to visible cell morphological alterations, although the changes were not as obvious as those observed in RON-overexpressing bladder cancer cells (Fig. 3H). Nevertheless, the altered cell morphologies were accompanied by increased expression of N-cadherin and Vimentin, the phenotypic marker of epithelial to mesenchymal transition (EMT) (Fig. 3I, J). In conclusion, results presented in Fig. 3 demonstrate that RON-mediated bladder cancer cell migration and invasion are channeled through MMP12. In other words, MMP12 is a downstream effector molecule that plays a key role in driving bladder cell cancerous migration and invasion.

### The role of HIF-2α in regulating MMP12 expression in bladder cancer cells overexpressing RON

**Fig. 4:**
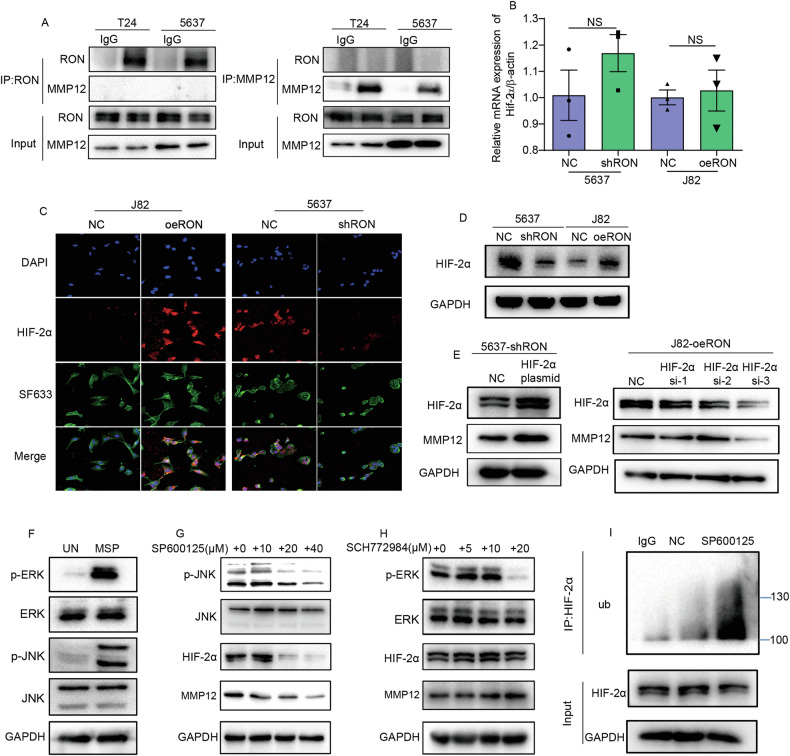
RON modulates the expression of MMP12 via inhibiting ubiquitination of the transcription factor HIF-2α. **A** Co-immunoprecipitation was employed to demonstrate the lack of direct interaction between RON and MMP12. **B**–**D** The mRNA expression levels of HIF-2α in 5637-shRON, 5637-NC, J82-oeRON, and J82-NC cells were quantified using quantitative real-time polymerase chain reaction (qRT-PCR), and confirmed by immunofluorescence staining and western blot analysis, respectively. **E** Transfection of 5637-shRON cells with a HIF-2α plasmid and J82-oeRON cells with HIF-2α-targeting siRNA was performed, followed by western blot analysis to assess the expression levels of HIF-2α and MMP12. **F** J82-oeRON cells were subjected to a 24-h period of starvation, followed by washing with ice-cold PBS to remove basal phosphorylation. Subsequently, the cells were stimulated with the RON-specific ligand MSP for 30 min, and western blot analysis was performed to assess the phosphorylation levels of ERK and JNK. **G**, **H** J82-oeRON cells were treated with varying concentrations of ERK inhibitors or JNK inhibitors for 24 h, after which the cells were harvested to evaluate the expression of HIF-2α and MMP12 by western blot. **I** J82-oeRON cells were subjected to treatment with or without 20 µM JNK inhibitors for a duration of 24 h, followed by the addition of 200 nM bortezomib 8 h prior to cell collection. Subsequently, the cells underwent immunoprecipitation using an anti-HIF-2α antibody, and the ubiquitination level of HIF-2α was assessed through immunoblotting with an anti-ubiquitin antibody. All experiments above were repeated three times (*n* = 3).

To elucidate the cellular relationships between RON and MMP12 in promoting bladder cancer cell invasive growth, we first study whether RON is physically in interaction with MMP12. Results from the Co-IP assays followed by the Western blot analysis among several cell lines tested did not find any evidence that RON directly interacts with MMP12. (Fig. 4A). Since HIF-2α is a direct transcription factor for MMP12 [20] and can be regulated by various types of kinase-independent of oxygen [21], we hypothesized that increased MMP12 expression in RON-overexpressing bladder cancer cells is mediated by RON-directed HIF-2α expression. To test this hypothesis, we first determined the HIF-2α mRNA levels in bladder cancer cells and found little changes in the levels of HIF-2α regardless of the amount of RON expressed by cancer cells (Fig. 4B). Surprisingly, the levels of the HIF-2α protein were strongly correlated with the amount of RON in bladder cancer cells (Fig. 4C, D). The probable explanation is that increased RON expression may stabilize HIF-2α in cellular compartments. Moreover, we found that in 5637-shRON cells that hardly express RON, the forced expression of HIF-2α can increase the MMP12 expression. Consistent with this finding, we also observed that in J82-oeRON cells that overexpressed RON, knockdown of HIF-2α expression resulted in diminished MMP12 (Fig. 4E). Taken together, these results suggest the presence of a potential signaling and functional axis among RON, HIF-2α, and MMP12 in the bladder cancer cells, which coordinately regulate bladder cancer cell migration and matrix invasion.

### The effect of the JNK signaling pathway on regulating RON-mediated cellular HIF-2α stability at the post-translational level

To understand how RON at the cell surface transduces signals leading to the intracellular accumulation of HIF-2α in bladder cancer cells, we focused our attention on JNK and ERK signaling pathways, both pathways have been implicated in regulating HIF-2α expression [22]. To this end, J82-oeRON cells overexpressing RON were serum-starved, washed, and then stimulated with MSP to activate the receptor and its downstream signaling pathways. The result in Fig. 4F showed that MSP-induced RON activation is accompanied by the increased phosphorylation of JNK and ERK in cancer cells. As expected, the RON-mediated activation of JNK and ERK was inhibited in the presence of JNK inhibitor SP600125 and ERK inhibitor SCH772984, respectively. An interesting finding was that the inhibition of JNK activity significantly reduces both HIF-2α and MMP12 expression. As evident in the results presented in Fig. 4G, obvious HIF-2α and MMP12 reduction were documented. The effect of ERK inhibitor SCH772984 on HIF-2α and MMP12 was not observed (Fig. 4H). We further observed that the inhibitor-mediated inhibition of the JNK signaling pathway results in increased ubiquitination of HIF-2α, which leads to a diminished HIF-2α level. As shown in Fig. 4I, the degree of ubiquitination of HIF-2a was significantly increased in comparison with that of control samples. To verify this observation, we performed additional studies using the RON activator and inhibitor in our cell models. Results in Fig. S2 showed that activation of RON in 5637 cells resulted in increased p-JNK, HIF-2α, and MMP12 expression. In contrast, inhibition of RON in J82-oeRON cells led to a reduction of HIF-2α expression, which is associated with decreased JNK activity and downregulation of MMP12 expression. Thus, results in Fig. 4 demonstrate that activation of RON, either by ligand stimulation or by overexpression, results in increased HIF-2α stability at post-translational level in bladder cancer cells. This effect at the signaling level is channeled through the activation of the JNK pathway, which negatively affects the cellular ubiquitination system to stabilize HIF-2α. The outcome of this chain activity is the increased MMP12 expression.

### Intriguing interaction of RON, specifically with microRNA hsa-miR-659-3p in bladder cancer cells

**Fig. 5:**
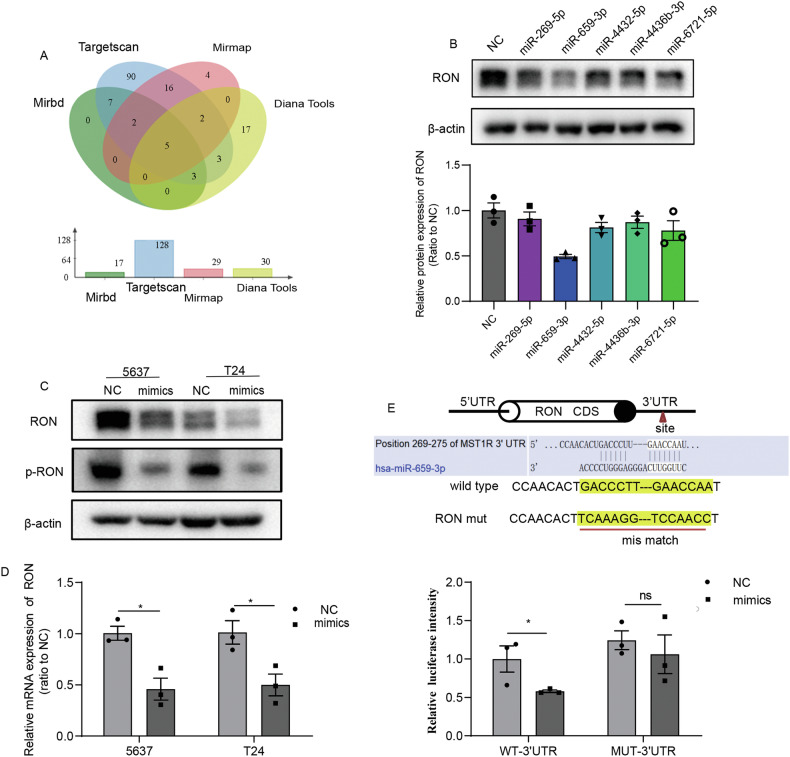
RON is identified as a target of hsa-miR-659-3p in bladder cancer cells. **A** A schematic illustrating the screening strategy employed to identify candidate microRNAs capable of regulating the expression of RON was presented. **B** The protein expression of RON in 5637 cells transfected with microRNA mimics or negative controls was assessed via western blotting. **C**, **D** The expression of RON and p-RON in 5637 and T24 cells transfected with miR-659-3p mimics or negative controls were evaluated using western blotting, and the expression of RON was further detected by quantitative real-time polymerase chain reaction (qRT-PCR). **E** A diagram illustrating the specific binding site of miR-659-3p within the 3′UTR region of RON mRNA, along with a mutated version of the RON 3′UTR, is presented. Luciferase reporter constructs containing either the wild-type or mutated RON 3′UTR were introduced into 293 T cells in conjunction with miR-659-3p mimics or control mimics, and luciferase activity was measured 24 h after transfection. The luciferase activity in cells transfected with control mimics was used as the reference point and normalized to a value of one. Experiments in **D**, **E** were repeated three times (*n* = 3).

The discovery of the RON > JNK > HIF-2α > MMP12 axis in promoting bladder cell tumorigenesis prompted us to ask how this pathway is intracellularly negatively controlled. To this end, we turned our effect to study whether certain microRNA species play a role in controlling RON expression and activity. A detailed analysis of putative miRNAs in interacting with RON using software programs including TargetScan, MiRDB, Mirmap, and Diana Tools led to several predictions that miRNAs such as miR-269-5p, miR-659-3p, miR-4432-5p, miR-4436b-3p, and miR-6721-5p might be able to interact with RON specifically (Fig. 5A). To prove these predictions, we exogenously overexpressed these 5 candidate miRNAs in 5637 cells and analyzed whether the cancerous RON expression is significantly affected. Results in Fig. 5B showed that among the 5 miRNAs tested, only miR-659-3p at the overexpression status was able to significantly reduce the RON expression. Additional studies using both 5637 and T24 cell lines further showed that increased miR-659-3p expression reduces the RON expression at both mRNA and protein levels (Fig. 5C, D). The downregulation of RON expression resulted in decreased RON phosphorylation (Fig. 5C). To understand how miR-659-3p regulates the RON expression in more detail, we inserted the RON 3’UTR containing a miR-659-3p target site and a mutant sequence lacking the target site into the downstream of a firefly luciferase coding sequence region. The generated reporter vectors were transferred into HEK 293 T cells simultaneously with either miR-659-3p mimics or control constructs. Results from analyzing the reporter expression indicated that miR-659-3p significantly inhibits the luciferase activities from the wild-type luciferase reporter vector with more than 40% reduction observed. No inhibitory effect was documented when the mutant miRNA lacking the target site was used (Fig. 5E). Thus, results from these studies confirm that the interaction of the RON mRNA with a particular miRNA exists intracellularly. The finding that RON was a specific target of hsa-miR-659-3p suggests that their intriguing interactions may hurt RON expression and function in bladder cancer cells.

### The negative effect of hsa-miR-659-3p on RON-mediated bladder cancer cell migration and invasion

**Fig. 6:**
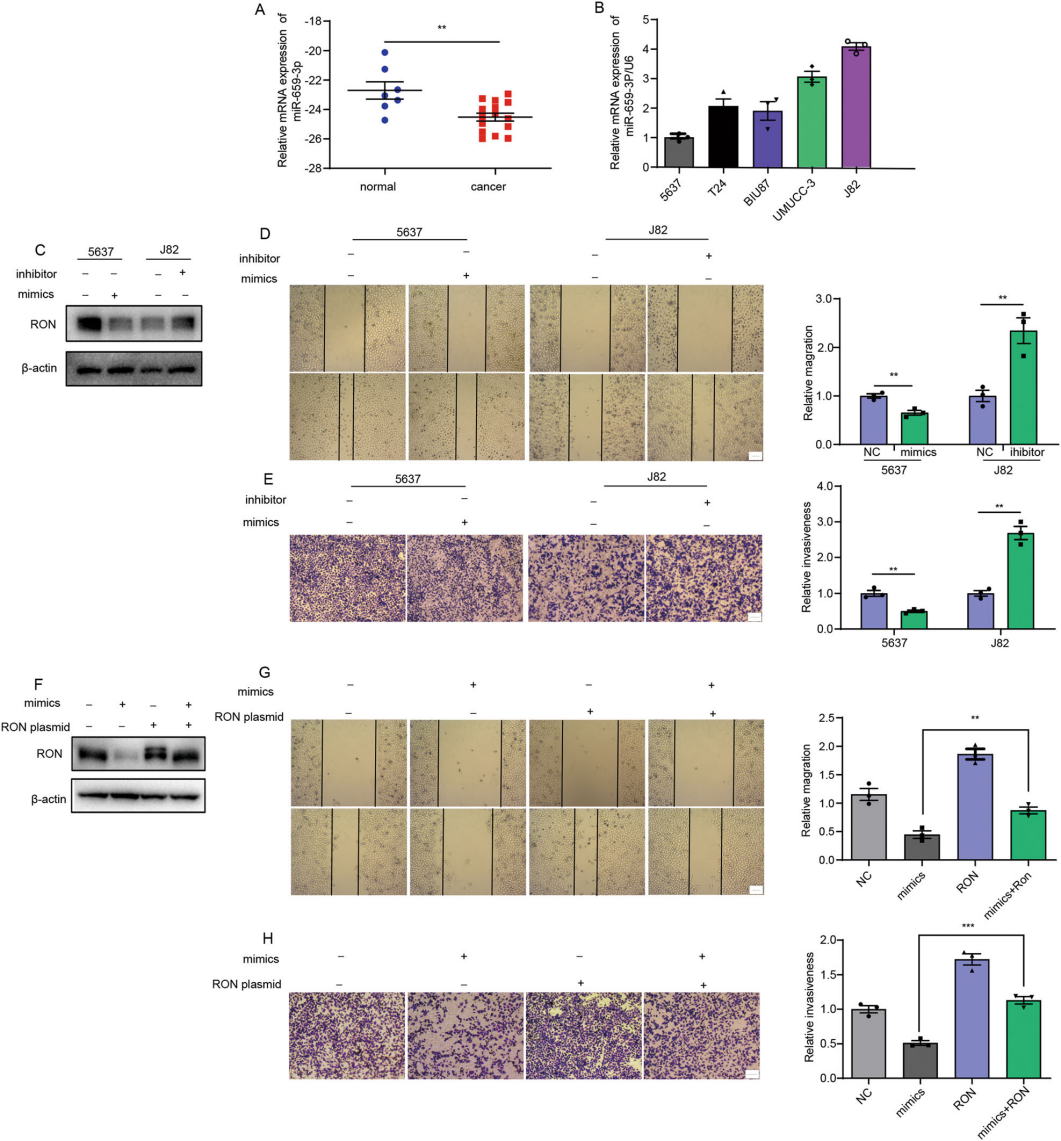
The detrimental impact of hsa-miR-659-3p on RON-mediated migration and invasion of bladder cancer cells. **A** The levels of miR-659-3p were assessed in normal bladder tissues (*n* = 7) and cancer samples (*n* = 15) using quantitative real-time polymerase chain reaction (qRT-PCR). **B** The expression of miR-659-3p was evaluated in various bladder cancer cell lines, with the 5637 cell line serving as the control. **C** Following transfection with miR-659-3p mimics or negative control in 5637 cells, and with miR-659-3p inhibitor or negative control in J82 cells, the expression of RON was confirmed through western blot analysis. **D** The migration ability was tested by wound-healing assay. **E** The invasion ability was evaluated by trans-well assay. (Scale bar 1 µm). **F** J82 cells were transduced with miR-659-3p mimics and a RON overexpression plasmid, followed by validation of RON expression using Western blot analysis. **G** The migration ability was tested by wound healing assay. **H** The invasion ability was evaluated by trans-well assay. (Scale bar 1 µm) Experiments in **B**–**H** were repeated three times (*n* = 3).

To study the role of miR-659-3p in RON-mediated bladder cell tumorigenesis, we first determined miR-659-3p expression in normal bladder tissues and cancer samples. A total of 22 samples were analyzed. The average levels of miR-659-3p expressed by cancer cells were significantly reduced in comparison with those from normal tissues (Fig. 6A). A test of the miR-659-3p expression in a panel of bladder cancer cell lines also confirmed that the expression levels of this particular miRNA were reversely correlated with the expression levels of RON (Fig. 6B). We next examined the effect of miR-659-3p on the migration and invasion of bladder cancer cells. As shown in Fig. 6D, E, 5637 cells transfected with miR-659-3p mimics demonstrated that this miRNA species can decrease bladder cancer cell migration and invasion in comparison with cells transfected with a scramble sequence. While J82 cells transfected with miR-659-3p inhibitor showed opposite results. Then, the effects of miR-659-3p mimics and inhibitors on RON expression have been confirmed in Fig. 6C. The findings were interesting, which encouraged us to determine whether miR-659-3p hurts RON-mediated tumorigenic activities in bladder cancer cells. In these cases, J82 cells transduced with miR-659-3p mimics together with the vector causing RON the overexpression was used for rescue experiments, and RON expression was reconfirmed in Fig. 6F. By overexpressing RON in bladder cancer cells, we observed that increased RON expression partially antagonizes the negative effect of miR-659-3p on RON-mediated cancer cell migration and invasion (Fig. 6G, H), indirectly supporting the negative role of miR-659-3p in RON expression and its associated function. Moreover, our findings indicate that miR-659-3p mimics indirectly affect the RON phosphorylation status in 5637 cells by downregulating RON expression, thereby diminishing the MSP-induced migratory and invasive activities (Fig. S3A, C, D). Furthermore, the miR-659-3p inhibitor-induced increase in migration and invasion, attributed to RON overexpression in J82 cells, can be mitigated by the effect of RON inhibitors (Fig. S3B, E, F). In summary, the results above demonstrate that by specific interaction with RON, hsa-miR-659-3p downregulates RON expression, which ultimately attenuates RON-mediated bladder cancer cell migration and invasion.

### BMS-777607, a RON-specific inhibitor, inhibits tumor growth and metastasis mediated by tumor cells overexpressing RON in animal models

**Fig. 7:**
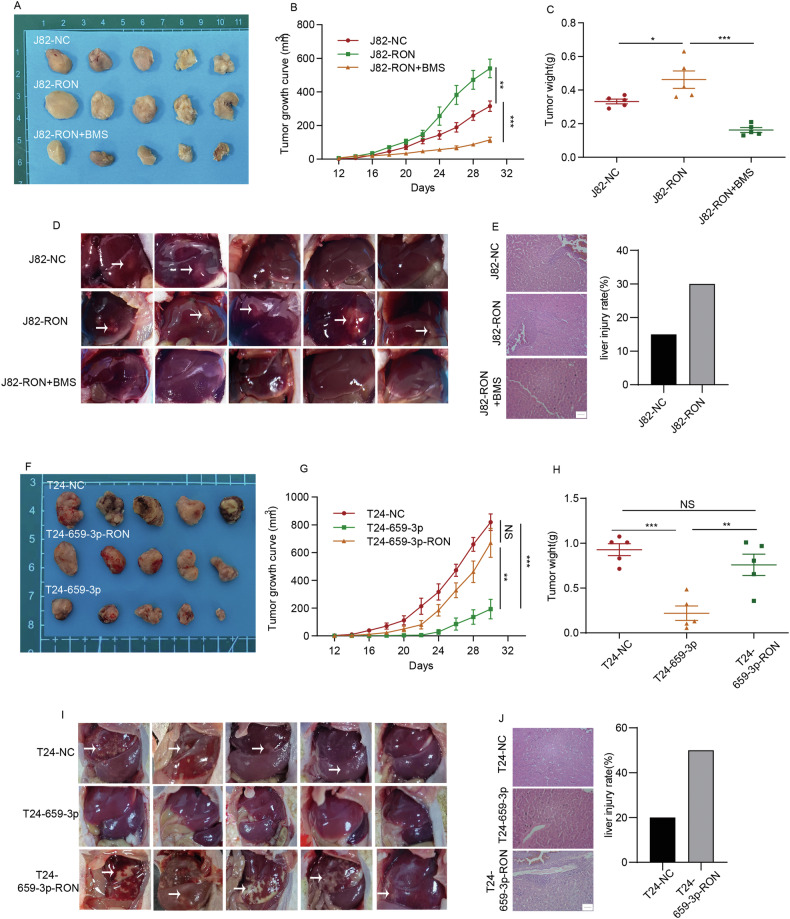
The inhibitory effect of BMS-777607 on RON-mediated activity and the suppressive effect of ectopic expression of miR-659-3p on bladder cancer cells tumorigenic in vivo. **A** J82 cells were infected with a lentivirus package to overexpress RON (J82-RON). An empty lentivirus vector package was used as a negative control to generate J82-NC cells. These two cell lines were inoculated into thymic nude mice, respectively, and tumor growth was monitored daily. Upon the tumor volume reaching ~100 mm^3^ (~14 days), mice were divided into 2 groups. Mice in the experimental group (J82-RON, 5 mice per group) were treated with BMS-777607 at 25 mg/kg/day. The mice in the control group (J82-RON, 5 mice per group) and mice from the J82-NC group, 5 mice per group) were administered with solvents. Images of tumors from individual mice were captured on day 32 following the injection of J82 cells. **B** The tumor growth was assessed daily and represented in a line chart. The weight of the tumor is shown in (**C**). **D** Upon the end of the study, all animals were dissected to confirm any organ metastases. Images of hepatic tissues were presented. **E** Hematoxylin and eosin (H&E) staining was performed to for pathological examination to find any metastatic tumors (Scale bar 500 nm). **F** T24 cells were transfected with LV-miR-659-3p to create T24-659-3p cells, which were subsequently transfected with lentivirus overexpressing RON to generate T24-659-3p-RON cells. The resulting T24 cells were then subcutaneously injected into the armpits of nude mice. Images of tumors from each group were captured on day 32 following the injection of transfected T24 cells. **G** The tumor growth was assessed periodically and represented in a line chart. The final weight of the tumor was shown in (**H**). **I** Then, all animals were further dissected to observe organ metastasis, and images of livers were illustrated. **J** Hematoxylin and eosin (H&E) staining was performed to detect metastatic livers. (Scale bar 500 nm).

Using in vitro cell models, we observed that BMS-777607 significantly inhibits tumor cell migration and invasion (Fig. 1J, K). To study the effect of BMS-777607 in vivo, J82 cells were infected with a lentivirus package to overexpress RON (J82-RON), while an empty vector lentivirus was used as a negative control (J82-NC). These cell lines were inoculated into thymic nude mice and tumor growth was monitored. BMS-777607 at 25 mg/kg was then injected into tumor-bearing mice (5 animals per group, tumor volumes at ~100 mm^3^). Mice without BMS-777607 treatment served as the control. As demonstrated in Fig. 7A–C, the average tumor size in mice injected with J82-RON cells was significantly larger than that from mice injected with J82-NC cells. Treatment of mice with BMS-777607 markedly inhibited tumor growth in the J82-RON-injected mice. These results suggest that increased RON expression results in increased tumor growth. This effect was inhibited after BMS-777607 treatment.

We further studied the effect of BMS-7778607 on tumor metastasis. Results in Fig. 7D showed that in mice injected with J82-NC cells, liver metastasis was observed in 2 out of 5 animals. In contrast, the hepatic lesion was observed in 5 out of 5 mice inoculated with J82-RON cells. Upon treatment of mice with BMS-777607, we did not observe liver metastasis in animals administered with J82-RON cells, which is confirmed by histological examination. In contrast, liver metastases were observed in both the J82-NC and J82-RON cell-injected mice, with the extent of hepatic damage being significantly greater in the J82-RON group compared to the J82-NC group (Fig. 7E). In summary, these results indicate that BMS-777607 effectively inhibits RON-mediated tumor growth and metastasis in animal models.

### The Inhibitory effect of forced miR-659-3p expression on the growth of tumor xenografts mediated by bladder cancer cells overexpressing RON

The in vivo study aimed to determine the effect of miR-659-3p on tumor growth was performed using T24 cell lines stably transfected with LV-miR-659-3p to overexpress miR-659-3p (T24-659-3p). These cells were also infected with a lentiviral package to overexpress RON (T24-659-3p-RON). The control cell line includes T24-NC. All cell lines were inoculated into the athymic nude mice (5 animals per group), and tumor growth was monitored accordingly. As shown in Fig. 7F–H, the average tumor size from T24-659-3p-injected mice was significantly smaller than that from the T24-NC-administrated animals. In contrast, the average tumor size from the T24-659-3p-RON group was comparable to that from the control group. These data indicate that the forced expression of miR-659-3p negatively affects the growth of bladder cancer xenografts and this effect was largely reversed by increased expression of RON. We then studied whether the forced MiR-659-3p expression affects tumor metastasis in mice. As shown in Fig. 7I, spontaneous tumor metastases in the liver at 80% (4/5) were observed in the mice injected with T24-NC cells. In contrast, no tumor metastasis was observed in animals administrated with T24-659-3p cells. Interestingly, tumor metastasis into the liver was the most serious (5/5) in mice inoculated with T24-659-3p-RON cells. Detailed histology analysis confirmed that there is no tumor metastasis in the liver in the T24-659-3p group. Metastases of tumors in the liver were observed in both T24-NC and T24-659-3p-RON cell-injected mice, and the degree of liver damage in the T24-659-3p-RON group was significantly higher than that in the T24-NC group (Fig. 7J). In conclusion, the results demonstrate that miR-659-3p is a negative regulator in vivo that significantly affects the kinetic growth of bladder cancer xenografts expressing RON. Increased RON expression can overcome the impact of miR-659-3p to increase the growth of bladder cancer xenografts in vivo.

## Discussion

The dysregulation of the RON gene product plays a crucial role in the pathogenesis of various cancers, including bladder cancer [8, 23]. Despite this understanding, the precise factors contributing to differential RON expression and the specific mechanisms involved in bladder cancer progression remain poorly unknown. This study aims to elucidate the significant role of RON in promoting migration and invasion in bladder cancer, as well as investigate the upstream and downstream mechanisms involved, providing new perspectives for the development of novel therapeutic strategies for bladder cancer.

In our prior clinicopathological data analysis, we observed a significant elevation of RON expression in bladder cancer tissues compared to adjacent tissues [11]. In the current study, we have identified a further significant increase in RON expression in invasive bladder cancer compared to non-invasive bladder cancer (Fig. 1A–C). To investigate the potential implications of RON in bladder cancer progression, wound healing, and trans-well assays were conducted on two bladder cancer cell lines. Our results indicate that increased RON expression enhances cell migration and invasiveness (Fig. 1H–K). These observations are consistent with those reported previously that aberrant RON expression and activation is a pathogenic factor contributing to the invasive phenotype of cancer cells. To gain a comprehensive understanding of RON in the malignant behaviors of bladder cancer cells, we applied cellular mRNA expression profiling techniques on 5637-shRON and 5637-NC cells. Our analysis revealed differential expression patterns between these two cell lines, with MMP12 identified as a downstream target of the RON signaling that is directly associated with invasive and migratory properties (Fig. 2).

Migration through the extracellular matrix is an essential component of cell migration, with matrix metalloproteinases (MMPs) playing a crucial role in degrading the matrix to promote tumor cell metastasis [24, 25]. Specifically, MMP12, a member of the MMP family, is associated with the metastatic phenotype in various cancers, including non-small cell lung cancer [26], hepatocellular carcinoma [27], squamous cell cancer [28], and osteosarcoma [29]. It is noteworthy that clinical studies have demonstrated an association between the polymorphism of the MMP12 G allele and an elevated risk of bladder tumor invasion and metastasis, attributed to the upregulation of MMP12 expression [30]. Our initial findings revealed that the downregulation of MMP12 effectively suppresses the cellular invasion and migration induced by RON overexpression in bladder cancer cells (Fig. 3). Moreover, Inhibition of MMP12 expression appeared to attenuate the RON-induced bladder cancer cell migration and invasiveness (Fig. S1). These results support the notion that MMP12 acts as an effector molecule in RON-mediated bladder cancer cell migration and matrix invasion.

To further investigate the specific mechanism of RON regulation of MMP12, we explored the potential direct interaction between RON and MMP12 in regulating its expression, however, our co-immunoprecipitation experiment yielded a negative result (Fig. 4A). Hypoxia serves as a significant microenvironmental influence that facilitates the metastasis of tumors. The clinical correlation between elevated expression levels of HIF-1α and HIF-2α and heightened rates of distant metastasis, as well as poorer overall survival outcomes, has been observed across various tumor types [31]. In addition to the well-established oxygen-dependent PHD/VHL/HIF-α regulatory pathway, there is growing interest in the role of oxygen-independent mechanisms in the modulation of HIF-α expression. Specifically, the impact of various kinases on HIF-α has been extensively documented [21]. However, the potential regulatory role of RON in HIF-2α through its kinase activity has not yet been documented in the literature. Previous research has shown that HIF-2α acts as a direct transcription factor for MMP12 [20], prompting further investigation into the potential regulatory role of RON in modulating MMP12 expression through its transcription factor HIF-2α. To investigate this hypothesis, we initially assessed the HIF-2α mRNA levels in bladder cancer cells and observed minimal fluctuations in HIF-2α levels irrespective of the RON expression levels in cancer cells (Fig. 4B). Interestingly, we noted a significant correlation between the levels of HIF-2α protein and the quantity of RON present in bladder cancer cells (Fig. 4C, D). This observation suggests that heightened RON expression may contribute to the stabilization of HIF-2α within cellular compartments. Furthermore, our study revealed that in 5637-shRON cells with low RON expression levels, the enforced expression of HIF-2α led to an increase in MMP12 expression. This observation was supported by our findings in J82-oeRON cells with high RON expression, where the suppression of HIF-2α expression resulted in a reduction in MMP12 levels (Fig. 4E). Collectively, the results presented in Fig. 4 indicate the existence of a potential signaling and functional axis involving RON, HIF-2α, and MMP12 in bladder cancer cells, which collectively govern the migration and invasion of these cells within the extracellular matrix.

To understand how RON at the cell surface transduces signals leading to the intracellular accumulation of HIF-2α in bladder cancer cells, we focused our attention on JNK and ERK signaling pathways, both pathways have been implicated in regulating HIF-2α expression [22]. Initially, we confirmed the capability of RON to activate the JNK and ERK signaling pathways, as depicted in Fig. 4F. Subsequently, our investigation revealed that suppression of JNK phosphorylation led to a reduction in HIF-2α and MMP12 protein expression, whereas inhibition of ERK phosphorylation did not yield similar outcomes (Fig. 4G, H). Concurrently, we observed that inhibition of JNK activity facilitates HIF-2α ubiquitination (Fig. 4I). Interestingly, RON activation in 5637 cells led to an increase in p-JNK expression, which consequently elevates the expression of HIF-2α and upregulated MMP12 expression. In contrast, inhibition of RON in J82-oeRON cells resulted in reduced HIF-2α expression, which is associated with JNK activity suppression and downregulation of MMP12 expression (Fig. S2). Taken together, these findings indicate that RON enhances the stability of HIF-2α at the post-translational level by activating the JNK signaling pathway, thereby facilitating the expression of MMP12.

The identification of the RON > JNK > HIF-2α > MMP12 axis in facilitating bladder cell tumorigenesis has led us to investigate the mechanisms by which this pathway is intrinsically regulated. In this regard, we have directed our focus toward examining the potential involvement of specific microRNA species in modulating RON expression and function. Alterations in miRNA have been detected in various types of human cancer, where they can function as oncogenes or tumor suppressors [32]. The expression profiles of miRNAs with potential as biomarkers can be utilized for the categorization, diagnosis, targeted treatment, and prognosis of diverse cancer types [15]. Nevertheless, there is a lack of published findings on miRNA research about RON in bladder cancer. In this study, we have identified RON as a target of has-miR-659-3p based on the luciferase reporter assay results (Fig. 5). Following the collection of clinical tissue samples, we utilized quantitative real-time polymerase chain reaction (qRT-PCR) to assess the expression of has-miR-659-3p. The findings indicated a down-regulation of has-miR-659-3p in bladder cancer tissues as opposed to normal tissues (Fig. 6A). This outcome implies that the reduction of has-miR-659-3p could potentially contribute to the elevated expression of RON in bladder cancer.

Previous studies have indicated that has-miR-659-3p plays a significant role in regulating the response to chemotherapy in colorectal cancer [33], the advancement of chronic myeloid leukemia [34], and the infiltration of bone marrow in stage M neuroblastoma [35]. However, limited research has been conducted on the involvement of has-miR-659-3p in other types of cancer. In the study, we found that has-miR-659-3p inhibits the migration and invasiveness of bladder cancer cells and this effect was dependent on the downregulation of RON, as evidenced by the restoration of cell migration and invasiveness following exogenous overexpression of RON (Fig. 6). Interestingly, the action of hsa-miR-659-3p directly decreases the RON expression, which indirectly affects the RON phosphorylation status, thereby inhibiting MSP-induced bladder cancer cell migration and invasion. Consistent with this observation, increased cell migration, and invasion were observed in cells treated with the hsa-miR-659-3p inhibitor, which can also be mitigated by specific RON inhibitor BMS-777607 (Fig. S3). Taken together, these results suggest that hsa-miR-659-3p exerts a negative regulatory effect on RON-mediated migration and invasion of bladder cancer cells.

Studies from in vivo models demonstrate that increased RON expression facilitates tumor growth and metastasis. This effect is sensitive to the inhibitory activity of BMS-777607. Additionally, it was observed that has-miR-659-3p suppressed tumor growth, a phenomenon that was counteracted by the overexpression of RON. Furthermore, has-miR-659-3p was found to impede tumor metastasis, although this inhibitory effect was exacerbated following RON overexpression (Fig. 7). The findings suggest that miR-659-3p serves as a potent negative regulator in an in vivo setting, exerting a substantial impact on the advancement of bladder cancer xenografts that express RON.

**Fig. 8:**
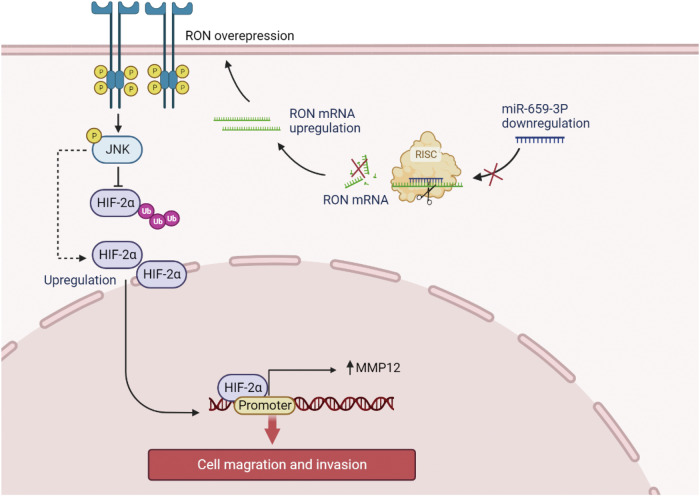
Illustration depicting the regulatory pathway involving hsa-miR-659-3p, RON, HIF-2α, and MMP12 in the context of bladder cancer cell migration and invasion. The inhibition of miR-659-3p resulted in the prevention of RON 3’UTR binding and subsequent RON mRNA degradation in the context of cancer progression. The overexpression of RON can lead to its activation through spontaneous phosphorylation, initiating downstream activation of the JNK signaling pathway, which in turn inhibits HIF-2α ubiquitination and stabilizes its expression. HIF-2α serves as a transcription factor for MMP12, promoting its expression and ultimately enhancing the migration and invasion of bladder cancer cells.

In summary, the current research has revealed that decreased expression of has-mir-659-3p plays a significant role in the upregulation of RON in bladder cancer. Furthermore, dysregulated RON has been implicated in the modulation of MMP12 expression through the activation of the JNK/HIF-2α signaling pathway, resulting in increased migration and invasion of bladder cancer cells. (Fig. 8). The identification of the hsa-miR-659-3p/RON and RON/JNK/HIF-2α/MMP12 pathways offer valuable insights into the pathogenesis of bladder cancer and may serve as potential targets for therapeutic interventions in the management of this disease.

## Methods

### Cell lines and reagents

Human bladder cancer cell lines, including 5637, T24, BIU87, UMUC-3, TCCSUP, and J82, were purchased from the Type Culture Collection of the Chinese Academy of Sciences (Shanghai, China)via certified short tandem repeat (STR) profiling. Tumorigenic features of these cell lines were described in detail on the provider’s website (www.cellbank.org.cn). Individual cell lines were cultured at 37 °C according to the provider’s instruction in their corresponding culture media containing 10% fetal bovine serum. Antibodies specific to MMP12 (ab137443) and β-actin (ab115777) were from Abcam (Cambridge, UK), antibodies specific to GAPDH (#2118), p-ERK (#4370), ERK (#4695), p-JNK (#4668), JNK (#9252), ubiquitin (#3936), and HIF-2α (#71565) were from CST (Boston, USA). Antibodies specific to p-RON and RON-specific ligand MSP were from R&D Systems (Minnesota, USA). BMS-777607, a specific RON inhibitor, was from Selleck (Selleck Chemicals, USA). The used antibodies include goat anti-rabbit IgG specific to HIF-2α, rabbit IgG, and mouse IgG1 (Cell signaling Technology, Boston, USA). Goat anti-MMP12 antibodies were from Abcam. RON (565692) was obtained from BD. The goat anti-rabbit IgG antibody coupled with horseradish peroxidase (HRP) was from Boster (Wuhan, China). Enhanced chemiluminescence reagents (ECLs) were from Sigma (St. Louis, Missouri, USA).

### Bladder tissue samples

All primary tumor and adjacent normal tissue samples were collected from oncological surgeries with the written consent of bladder cancer patients administrated in the First Affiliated Hospital of Ningbo University (Ningbo, China). The protocol of using human tissues for biological analysis (#2022-065A) was approved by the Ethical Committee of the First Affiliated Hospital of Ningbo University.

### Quantitative real-time PCR (qRT-PCR)

Total RNA was extracted from bladder cancer cells using TRIzol reagents (Invitrogen, United States), followed by cDNA synthesis using a reverse transcription kit (Thermo, USA). The qRT-PCR method was performed using SYBR Green PCR Master Mix (Roche, USA) on a LightCycler480 system with several primers (Table S2). The relative abundance of the DNA contents was determined using the ∆Ct method.

### Cell migration and matrix invasion assay

The cell migration assay was performed using the wound healing assay as previously described [36]. Bladder cancer cells were cultured to reach ~95% confluence, followed by creating a linear wound across the diameter of the well. Cells were allowed to move into the open space for up to 18 h. Photographic images were taken from individual wells, and levels of cell migration were quantitatively measured and analyzed as previously described. The matrix invasion assay was carried out using trans-well chambers (8 mm pore diameter) as previously described. Cells at 2 × 10^4^ per well in a serum-free media were seeded for 48 h in the upper chamber with filters coated with BD Matrigel. Cells invaded through the pore were fixed with paraformaldehyde, stained with crystal violet, followed by decolorized using glacial acetic acid. Color intensities from individual samples were quantitatively measured at 570 wavelengths. The obtained values were converted into the cell number according to a standard curve.

### RNA sequencing

Total RNA was extracted using TRIzol reagent (Invitrogen, Germany) and sequenced at Aksomics Technology (Shanghai, China). Independent triplicate samples were used for sequencing. The RNA sequencing data were submitted to the Sequence Read Archive (SRA) data under accession number PRJNA1184403.

### Co-immunoprecipitation (Co-IP) and Western blotting

The Co-IP assay was performed according to the previously described [37]. Cellular proteins were prepared by lysing cells in the RIPA lysis buffer containing phenyl-methanesulfonyl fluoride (PMSF) and protease inhibitor (Solarbio, Beijing, China) and then mixed with specific antibodies followed by the addition of 30 µl of protein G Sepharose beads (#sc-2003, Santacruz, Shanghai, China). The isotype-matched IgG was used as the control. After repeated washing, the collected immunoprecipitates were suspended in the 2× SDS sample buffer, separated un in 10% SDS-PAGE under reduced conditions. Western blot analysis was performed as previously described [38]. Immunoprecipitants or cellular proteins at 20 µg per sample per lane from individual cell lysates were separated in a 10% SDS-PAGE under the reduced condition and then transferred to a PVDF membrane. Specific proteins were detected using their corresponding antibodies, followed by the second antibody coupled with HRP, visualized with enhanced chemiluminescent reagents, and then recorded in the Bio-Rad Image system.

### Cellular immunofluorescence staining

Bladder cancer cells were fixed in 4% (w/v) paraformaldehyde, washed with PBS, and then permeabilized with 0.1% Triton X-100 as previously described [39]. Cells were first incubated overnight with goat anti-HIF-2α antibody (1:200 dilution) followed by the incubation with the goat anti-rabbit IgG coupled with Alexa Fluor® 555 78 (Abcam, city, USA) for 60 min. After repeated washing, phalloidins (Alexa Fluor® 630, Solarbio, Beijing, China) were added according to the manufacturer’s instructions. In addition, DAPI fluorescent dye (1 µg/ml) was added to each sample. at the end of the assay, all samples were photographed and analyzed using confocal scanning microscopy (NOVEL, Ningbo, China).

Generation of stable bladder cancer cell lines overexpressing/down-expressing RON and their corresponding negative control variants: Lentiviral constructs containing specific sequences for overexpressing or silencing RON were from Genechem (Genechem, Shanghai, China). The lentivirus packaging for the RON overexpression contains 3 components: plasmid GV492 carrying RON coding sequence region, plasmid helper (pHelper)-1.0, and plasmid helper (pHelper)-2.0. The lentivirus packaging for the RON knockdown also contains 3 plasmids as described above. The only exception is plasmid GV492, which harbors a short hairpin RNA oligonucleotide sequence (shRNA) for silencing the RON gene expression. The shRNA sequence is 5-CCGGGAGGTCAAGGATGTGCTGATTCTCGAGAATCAGCACATCCTTGACCTCTTTTT-3. A control plasmid containing a scramble shRNA was also used. To establish stable RON-overexpressing cell lines, the bladder cancer J82 cells at 50% confluence in a 6-well plate were infected with the lentiviral package according to the manufacturer’s instruction (https://www.genechem.com.cn/proinfo/16.html). The obtained cell lines were designated as (J82-oeRON). Similar procedures were also used to establish the stable RON-negative control cell line (J82-NC). For generating stable RON knockdown cell lines, bladder cancer 5637 cells were infected with the lentiviral packages containing the RON-specific shRNA to produce a stable cell line (5637-shRON). Again, a negative control cell line (5636-NC) was generated using the constructs harboring the scramble sequence. Expression of RON in these cell lines was confirmed by Western blot analysis using antibodies specific to human RON.

### Establishment of transient cancer cell lines

For generating cell lines transiently expressing transfected with siRNAs, overexpression plasmids, microRNA mimics, and microRNA inhibitors using Lipofectamine™ 3000 reagent (Thermo Fisher Scientific, Massachusetts, USA) according to the manufacturer’s instruction. The cells were harvested after a 48 h incubation to determine the transfection efficiency. The sequence of hsa-miR-296-5p, hsa-miR-659-3p, hsa-miR-6731-5p, hsa-miR-4436b-3p, hsa-miR-4632-5p mimics, hsa-miR-659-3p inhibitors, MMP12 siRNA, and their corresponding controls are shown in Table S1. Mammalian expression vectors containing the full-length human RON and MMP12 cDNA were purchased from Genechem (Shanghai, China).

### Generation of stable bladder cancer cell lines expressing miRNAs

hsa-miR659-3p-expressing lentivirus with luciferase and puromycin resistance markers were purchased from Genechem company (Shanghai, China). GV303 pHelper1.0 and pHelper2.0 vectors were used to package the lentivirus. T24 cells were infected with hsa-miR659-3p-expressing lentivirus (termed as T24-659-3p). Next, we constructed a new RON-overexpression lentivirus using the P14038 plasmid (main component information: pCDH-CMV-MCS-EF1-Blast) as well as the two helper plasmids mentioned earlier. After a stable cell line was obtained by screening with 2 μg/ml puromycin, T24-659-3p cells were infected with RON-overexpression lentivirus (termed as T24-659-3p-RON) and continued screening with 2 μg/ml puromycin and 10 μg/ml Blasticidin S. T24-NC cells harbored both sets of control lentivirus mentioned above.

### Tumor xenografts in mouse model

All procedures used in animal studies (IACUC protocol #20010178) have been approved by the Ningbo University Institutional Animal Care and Use Committee. A total of five mice were used in each group, and no blinding methods were used. The details are as follows: for the J82 cell model, athymic BALB/c-nu mice (male, aged at 5 weeks, from China Vital River Co, City, country) were randomly divided into 3 groups (5 animals per group). J82-NC, J82-RON, and J82-RON cell lines at 1 × 10^7^ cells per mouse in 25 µl PBS, respectively, were injected subcutaneously into the animal’s armpit. After 14 days, Mice with tumors from J82-RON cells were treated with BMS-777607 at 25 mg/kg/day (J82-RON + BMS). The remaining tumor-bearing mice from J82-RON cells and animals from J82-NC cells served as the control group. For the T24 cell model, athymic BALB/c-nu mice (male, aged at 5 weeks) were from China Vital River Co (City, country) and randomly divided into 3 groups (5 animals per group). Bladder cancer cell lines T24-NC, T24-659-3p, and T24-659-3p-RON at 1 × 10^7^ cells per mouse in 25 µl PBS, respectively, were injected subcutaneously into the animal’s armpit. Tumor growth was monitored every 2 days by measuring individual tumor volumes using the formula: *V* = *L* × W2 × *π*/6 (*V*, volume; *L*, length; and *W*, width of tumor) as previously described [38]. At day 32, all mice were euthanized accordingly and tumor xenografts were harvested from animals to be examined for any metastatic lesions in tissue and organs. Some tumor samples and organs were also fixed and hematoxylin-eosin (H&E) was stained to pathologically confirm tumor appearances and possibilities of metastasis.

### Statistical analysis

All statistical analyses were performed using Prism v9.0 software (GraphPad, USA). The data in all figures are shown as means ± SEM. The chi-square test was used to determine the statistical differences of results in Fig. 1B, C. ANOVA was used to evaluate the differences between groups. A two-sided Student’s *t*-test was used to compare the differences between the two groups. Differences were considered significant if *p* < 0:05, ^∗^*p* < 0:05, ^∗∗^*p* < 0:01, and ^∗∗∗^ *p* < 0:001.

## Supplementary information

Supplementary Figures

Supplementary tables

wb original data

## Data Availability

The data in this study are available upon request from the corresponding author.
