# Supplementary material for: RON receptor tyrosine kinase as a critical determinant in promoting tumorigenic behaviors of bladder cancer cells through regulating MMP12 and HIF-2α pathways
Source: Cell Death Dis. 2024 Nov 19;15(11):844. doi: 10.1038/s41419-024-07245-w (PMC11574271; doi:10.1038/s41419-024-07245-w)
Supplement: Supplementary file 1 — Supplementary Figures [file 41419_2024_7245_MOESM1_ESM.rtf]

Fig. S1 The effect of differential MMP12 expression on migration and invasion of bladder cancer cells with different levels of RON expression. 
(A)5637 cells were treated with 5nM MSP and then transfected with siRNAs targeting MMP12. Western blot analysis was performed to confirm MMP12 expression. (B) Cell migration was determined by using the wound-healing assay. (C) The cell invasive activity was determined by using the trans-well assay. (D) J82-oeRON cells were treated with 5 μM BMS-777607 for 24 hours and then transfected with an MMP12 expression vector. Western blot analysis was performed to confirm MMP12 expression. (E) Cell migration was determined by using the wound-healing assay. (F) Cell invasive activity was determined by using the trans-well assay. All experiments above were repeated three times(n=3).


Fig. S2 Regulation of RON expression and activity affects MMP12 expression via the JNK/HIF-2α signaling pathway.
(A)5637 cells were treated with 5 nM MSP. J82-oeRON cells were treated with 5μM BMS777-607. After incubation for 24 hours, cells were collected, lysed, and subjected to Western blot analysis.  blot analysis to determine the levels of p-RON, p-JNK, HIF-2α, and MMP12.The experiments above were repeated three times(n=3).


Fig. S3 The effect of miR-659-3p on the RON phosphorylation status and expression levels and their impact on RON-mediated cell migration and invasion.
(A) 5637 cells were treated with 5 nM MSP and then transfected with miR-659-3p mimics. Western blot analysis was performed to determine RON expression and phosphorylation status. (B) Cell migration was determined by using the wound-healing assay. (C) Cell invasive activity was determined by using the trans-well assay. (D) J82 cells were treated with 5 μM BMS-777607 and then transfected with miR-659-3p inhibitors. Western blot analysis was performed to determine RON expression and the phosphorylation status (p-RON). (E) Cell migration was determined by using the wound-healing assay. (F) Cell invasive activity was determined by using the trans-well assay. All experiments above were repeated three times(n=3).
