# Supplementary material for: RON receptor tyrosine kinase as a critical determinant in promoting tumorigenic behaviors of bladder cancer cells through regulating MMP12 and HIF-2α pathways
Source: Cell Death Dis. 2024 Nov 19;15(11):844. doi: 10.1038/s41419-024-07245-w (PMC11574271; doi:10.1038/s41419-024-07245-w)
Supplement: Supplementary file 2 — Supplementary tables [file 41419_2024_7245_MOESM2_ESM.docx]

**Table S1.** **The sequences of miRNAs’ mimics, inhibitors, MMP12 siRNA and HIF-2α** **siRNA.**

| Name of the sequence | Sense (5'-3') | Antisense (5'-3') |
| --- | --- | --- |
| Mimics NC | UUCUCCGAACGUGUCACGUTT | ACGUGACACGUUCGGAGAATT |
| miR-296-5p mimics | AGGGCCCCCCCUCAAUCCUGU | AGGAUUGAGGGGGGGCCCUUU |
| miR-659-3p mimics | CUUGGUUCAGGGAGGGUCCCCA | GGGACCCUCCCUGAACCAAGUU |
| miR-6721-5p mimics | UGGCAGGGGCUUAUUGUAGGAG | CCUACAAUAAGCCCCUGCCCAUU |
| miR-4436b-5p mimics | CAGGGCAGGAAGAAGUGGACAA | GUCCACUUCUUCCUGCCCUGUU |
| miR-4632-5p mimics | GAGGGCAGCGUGGGUGUGGCGGA | CGCCACACCCACGCUGCCCUCUU |
| Inhibitor NC | CAGUACUUUUGUGUAGUACAA | |
| miR-659-3p inhibitor | UGGGGACCCUCCCUGAACCAAG | |
| Control | UUCUCCGAACGUGUCACGUTT | ACGUGACACGUUCGGAGAATT |
| MMP12 si-1 | GCCUUGAGAUAAACAAACUTT | AGUUUGUUUAUCUCAAGGCTT |
| MMP12 si-2 | CACCUACAGAAUCAAUAAUTT | AUUAUUGAUUCUGUAGGUGTT |
| MMP12 si-3 | GAGCCAAAUUAUCCCAAGATT | UCUUGGGAUAAUUUGGCUCTT |
| HIF-2α si-1 | GGAGCUAACAGGACAUAGUTT | ACUAUGUCCUGUUAGCUCCTT |
| HIF-2α si-2 | GCGCAAAUGUACCCAAUGATT | UCAUUGGGUACAUUUGCGTT |
| HIF-2α si-3 | CGCUCAGCCUAUGAAUUCUTT | AGAAUUCAUAGGCUGAGCGTT |

**Table S2. Oligonucleotide sequence of primer set used to amplify in each cDNA.**

| Gene | Forward primer (5'-3') | Reverse primer (5'-3') |
| --- | --- | --- |
| β-actin | AAACGTGCTGCTGACCGAG | TAGCACAGCCTGGATAGCAAC |
| RON | GCAGTGGAAAGCAGGTGTGAG | CGAAAGCCAGGCAGTGTAAAG |
| PLAT | ACTCAGTGCCTGTCAAAAGT | CACGTGGCCCTGGTATCTATT |
| ASAP3 | CTCTTTCCTTGGGGTCTAGGG | CCCCGGCTTTGAGCAGATAC |
| GRB7 | GCTCGGGGTTCAGGACAAG | CTCCTCAGGCAGAGGGGTAT |
| PARD6A | ATACGGATGCTCATGGCGAC | GTCAGCTTCTGCCCGCTTCT |
| MDK | GAGTCGCCTCTTAGCGGATG | GCCGCCCTTCTTCACCTTAT |
| NTN4 | ATGCTTGCAAACCGTGTTCC | GTCGACAGCCATAGTCTCCG |
| SEMA4A | GATGGGGTTGAGAATGGGGG | GAGCTCTAGGCCAGGGAAAC |
| PTP4A3 | ACACATGCGCTTCCTCATCA | GTCACTTCACACACACGCAC |
| NRCAM | ACAACTGTGGATGAAGCTGGT | AGCAGCTGCAGCAGTAAGAT |
| DCHS1 | TGCATGTTGCAGTGCTTGAC | CCTCATCTGGATCCTTCGCC |
| MMP12 | TTTGGTGGTTTTTGCCCGTG | TCGAAATGTGCATCCCCTCC |
| FGFR4 | TTCCGGCAAGTCAAGCTCAT | TTCCCAAGCACCAGCGAG |
| HIF-2α | CCTTCCGACTCCCAGCATTC | GAGGCTGTCAGACCCGAAAA |
| U6 | CAGCACATATACTAAAATTGGAACG | ACGAATTTGCGTGTCATCC |
| MiR-659-3p | GATGTTCACTTGGTTCAGGGA | CTCTGTCTCTCGTCTTGTTGGTAT |
