# Supplementary material for: RON receptor tyrosine kinase as a critical determinant in promoting tumorigenic behaviors of bladder cancer cells through regulating MMP12 and HIF-2α pathways
Source: Cell Death Dis. 2024 Nov 19;15(11):844. doi: 10.1038/s41419-024-07245-w (PMC11574271; doi:10.1038/s41419-024-07245-w)

Figure1

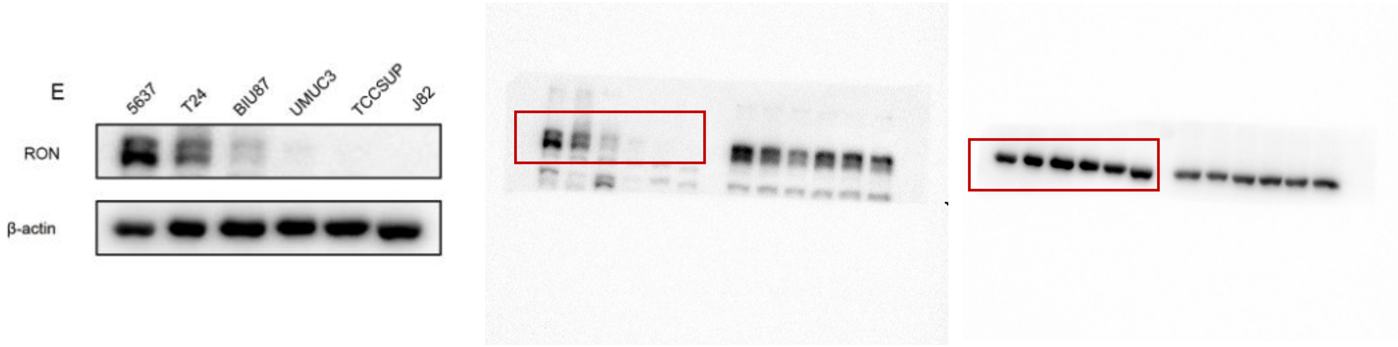
E
5637
T24
BIU87
UMUC3
TCCSUP
J82
RON
β-actin

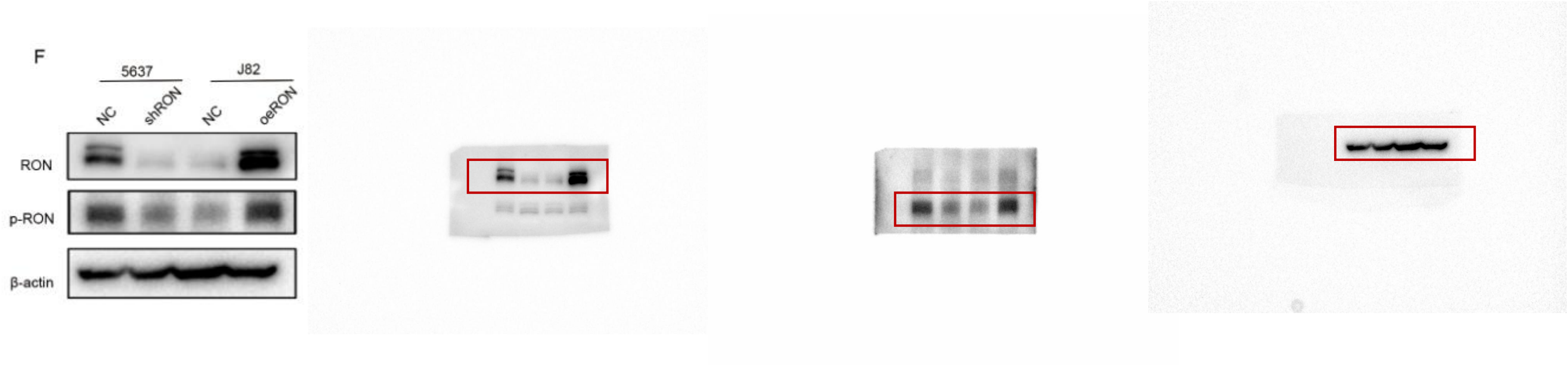
F
5637
J82
NC
shRON
NC
oeRON
RON
p-RON
β-actin

Figure1

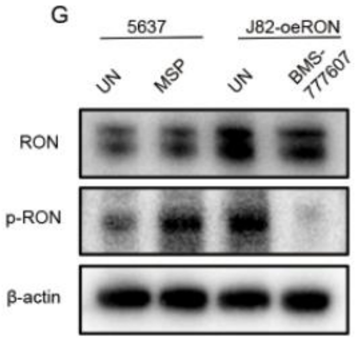

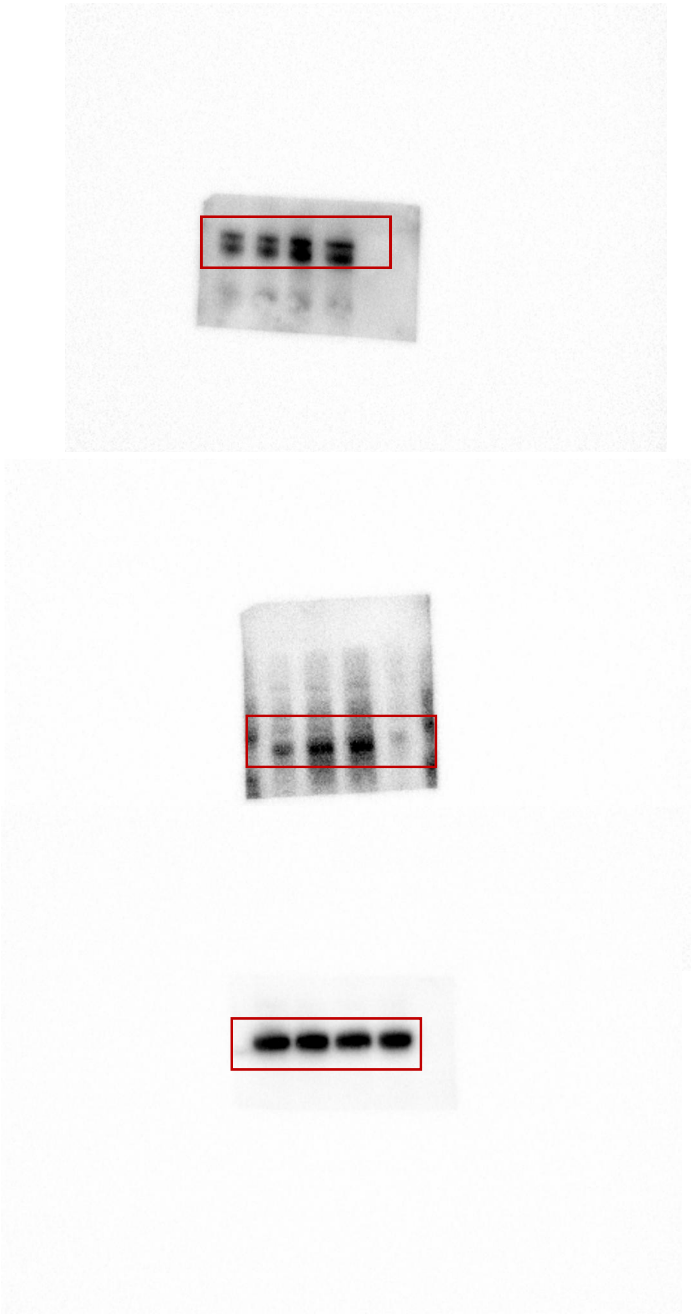

Figure2

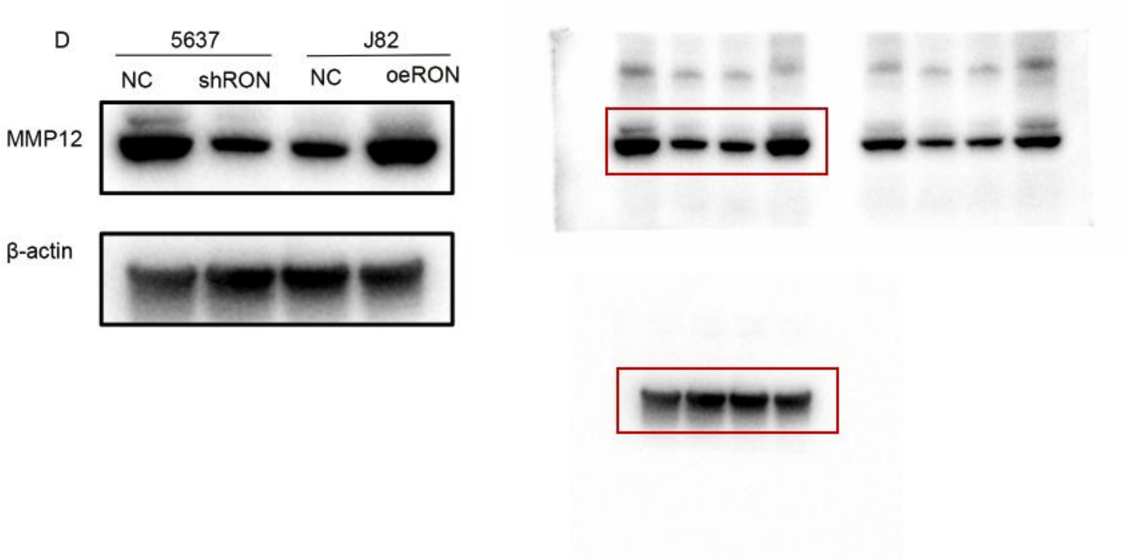

Figure3

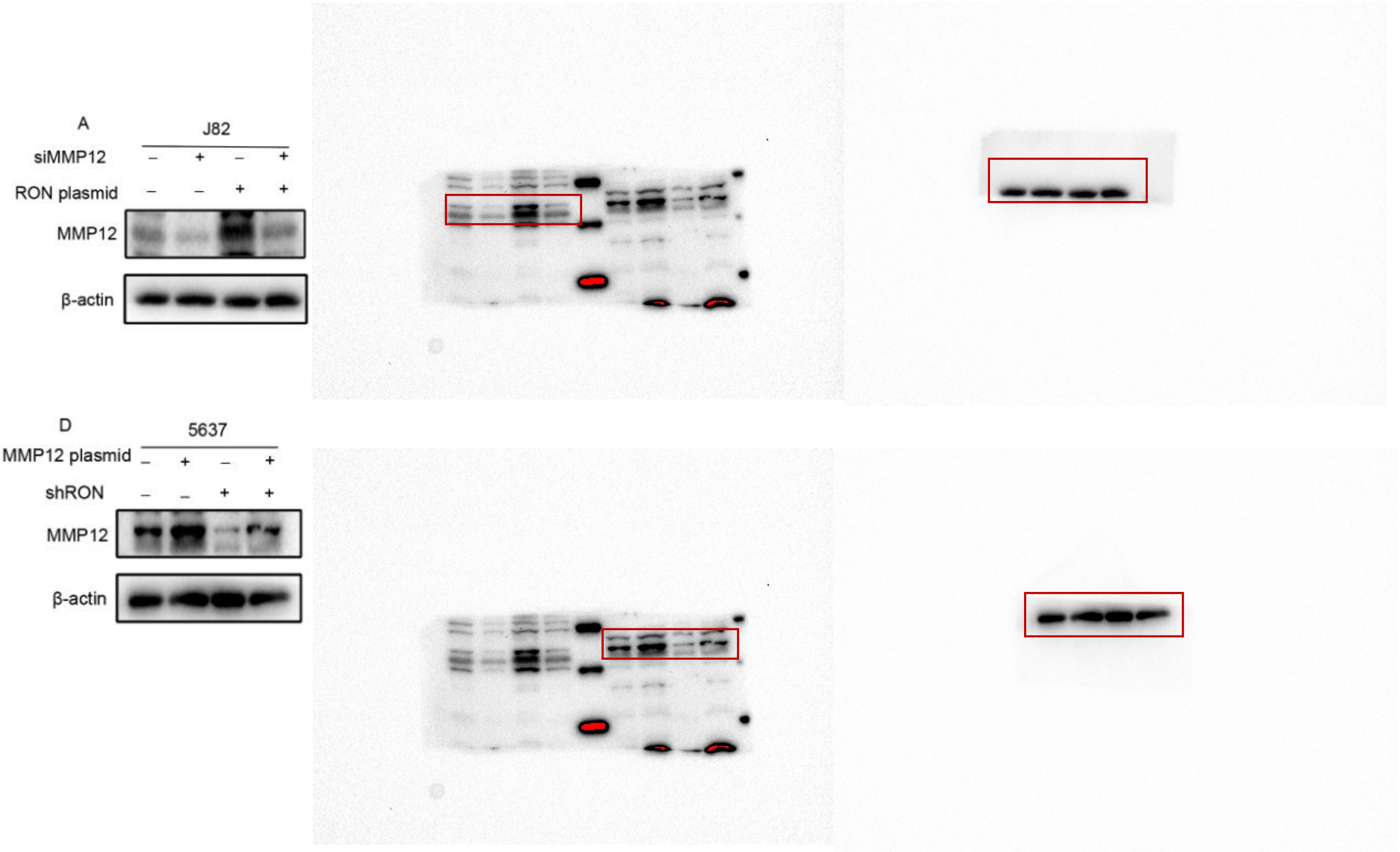

A
J82
siMMP12 – + – +
RON plasmid – – + +
MMP12
β-actin
D
5637
MMP12 plasmid – + – +
shRON – – + +
MMP12
β-actin

Figure3

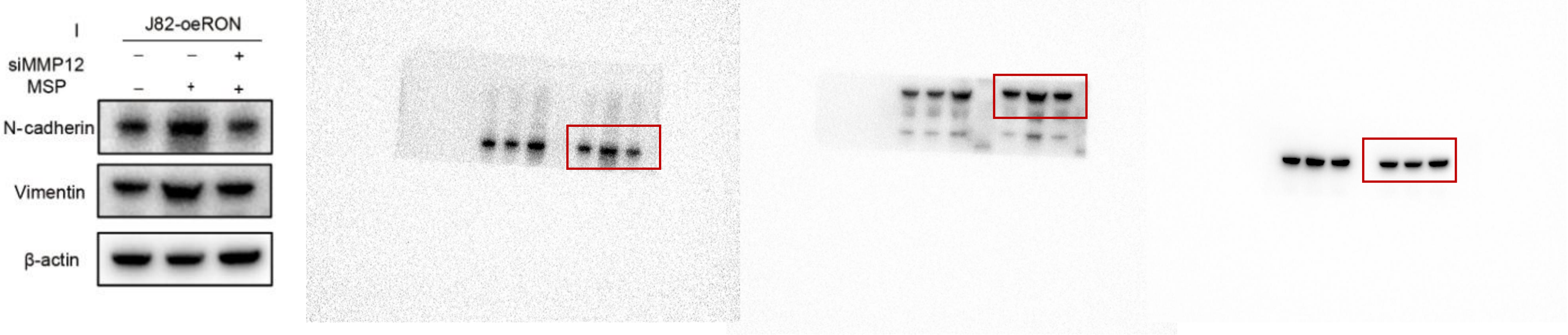
I
J82-oeRON
siMMP12 – – +
MSP – + +
N-cadherin
Vimentin
β-actin

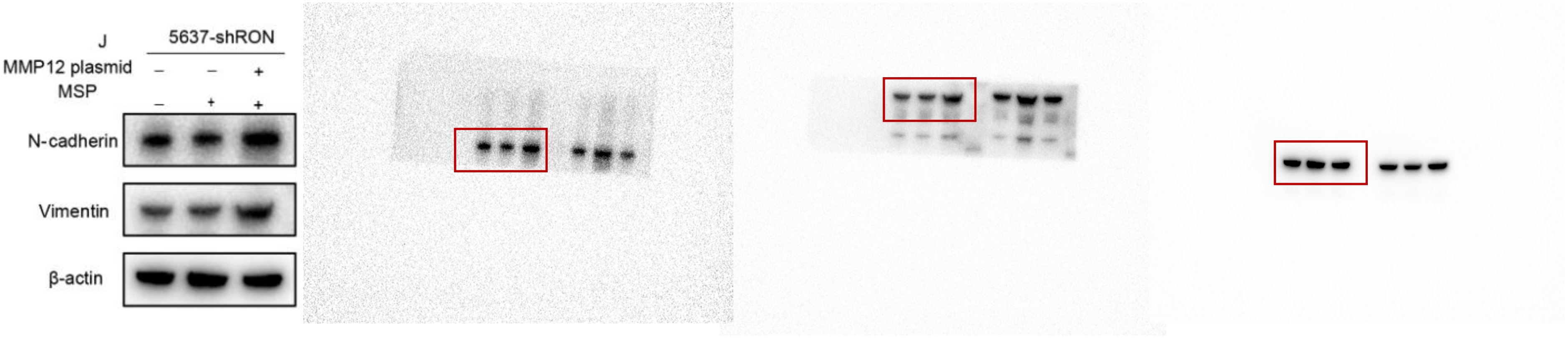
J
5637-shRON
MMP12 plasmid – – +
MSP – + +
N-cadherin
Vimentin
β-actin

Figure 4

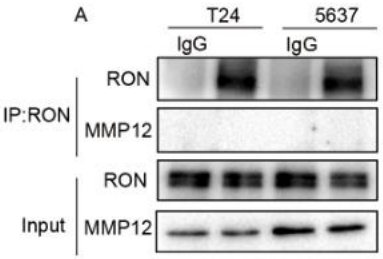
A
T24
5637
IgG
IgG
IP:RON
RON
MMP12
Input
RON
MMP12

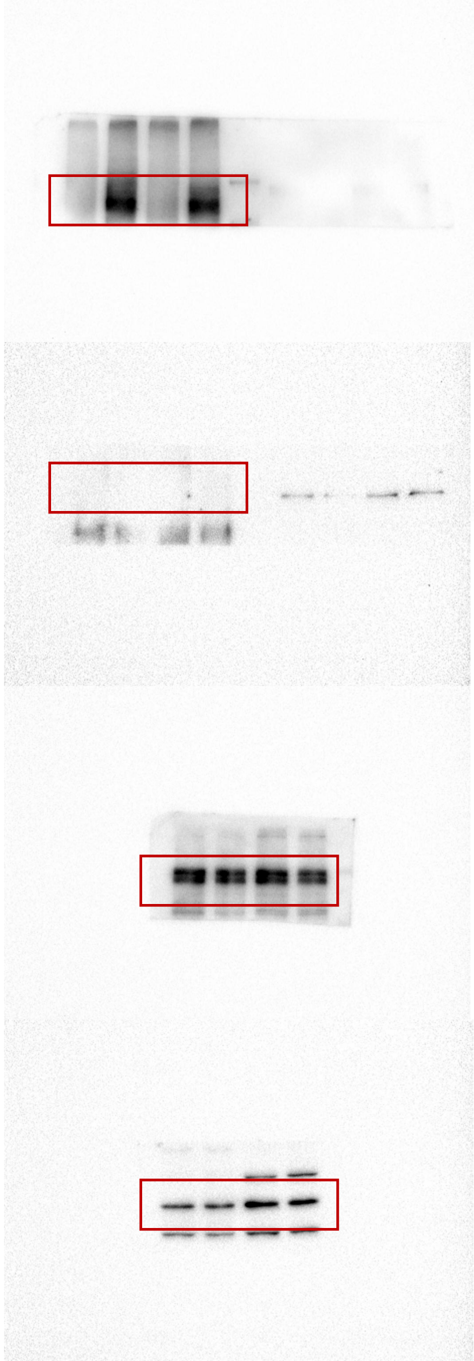

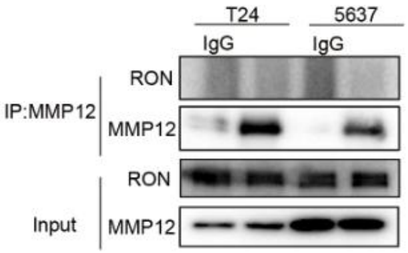
T24
5637
IgG
IgG
IP:MMP12
RON
MMP12
Input
RON
MMP12

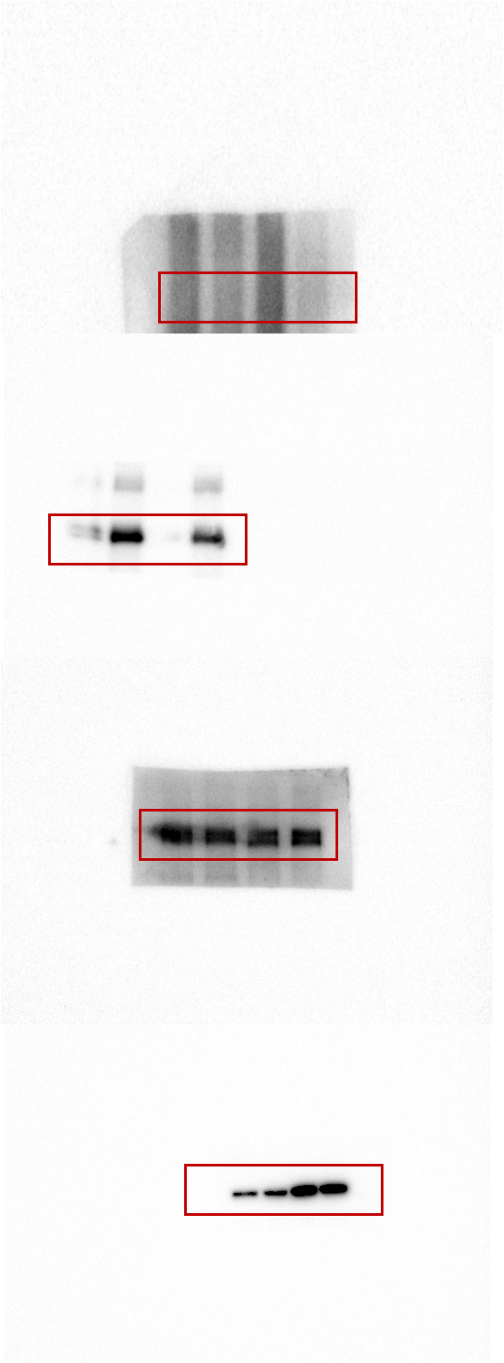

Figure 4

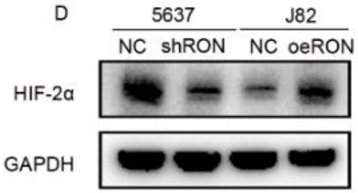

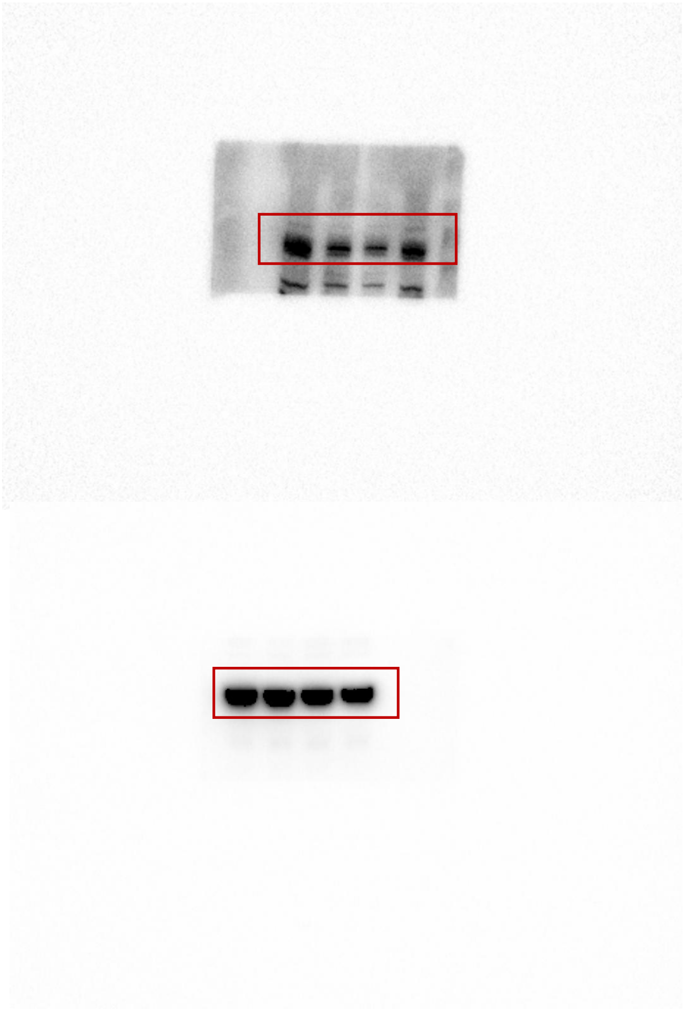

Figure 4

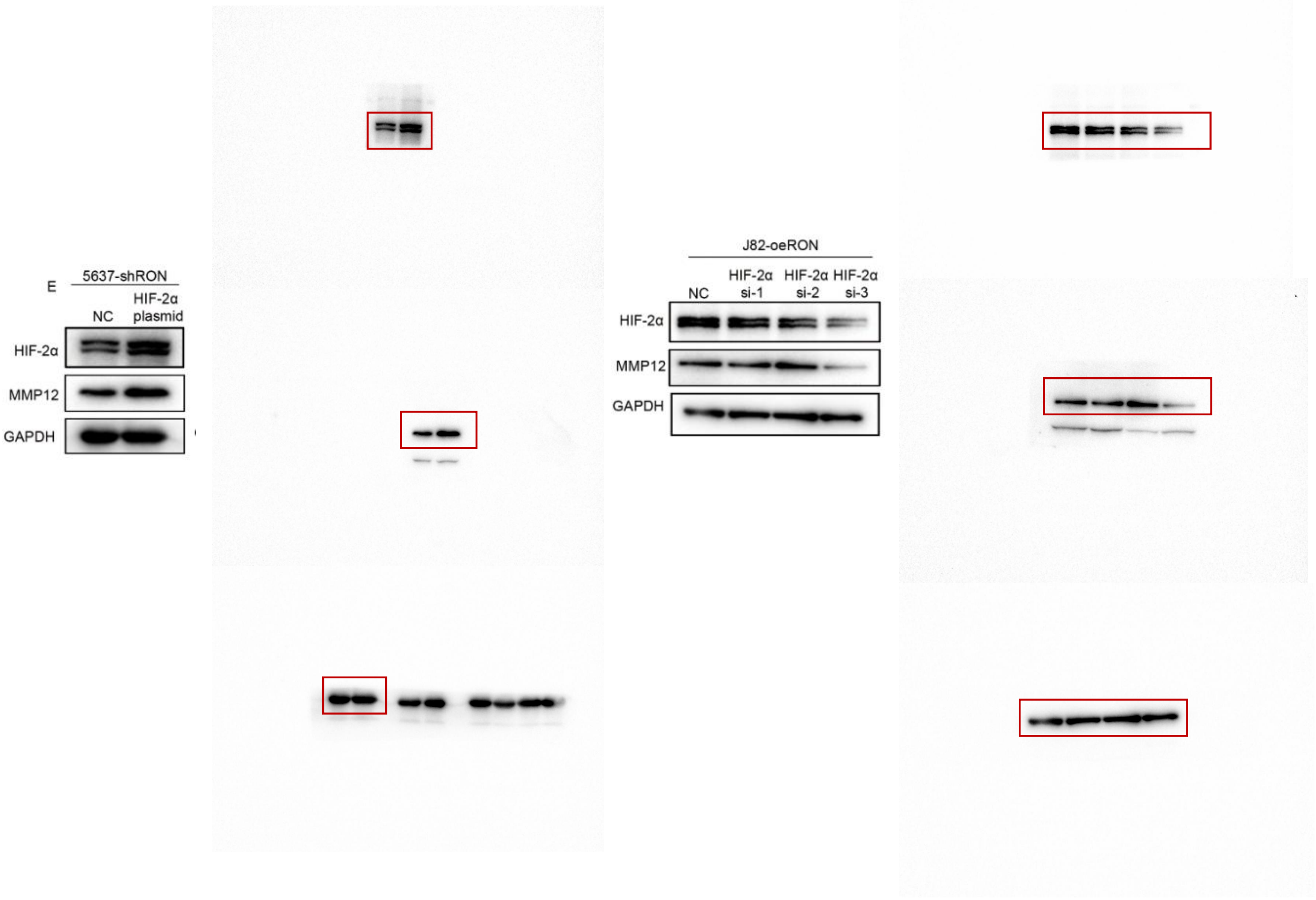

E
5637-shRON
NC
HIF-2α plasmid
HIF-2α
MMP12
GAPDH
J82-oeRON
NC
HIF-2α si-1
HIF-2α si-2
HIF-2α si-3
HIF-2α
MMP12
GAPDH

Figure 4

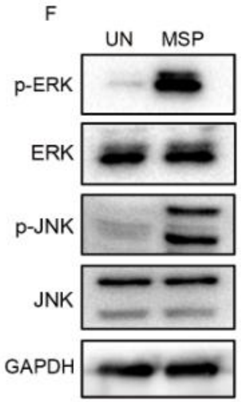
F
UN
MSP
p-ERK
ERK
p-JNK
JNK
GAPDH

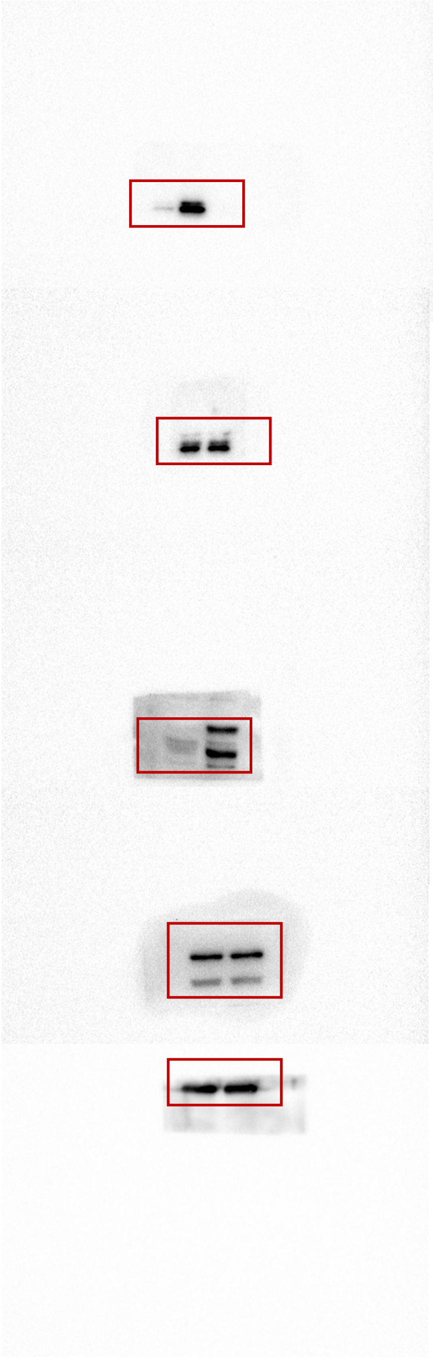

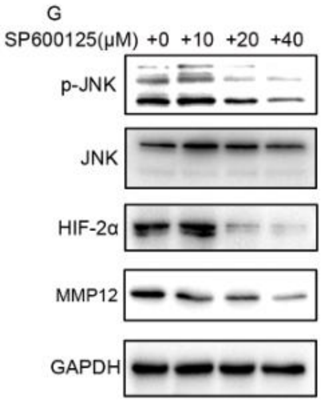
G
SP600125(μM) +0 +10 +20 +40
p-JNK
JNK
HIF-2α
MMP12
GAPDH

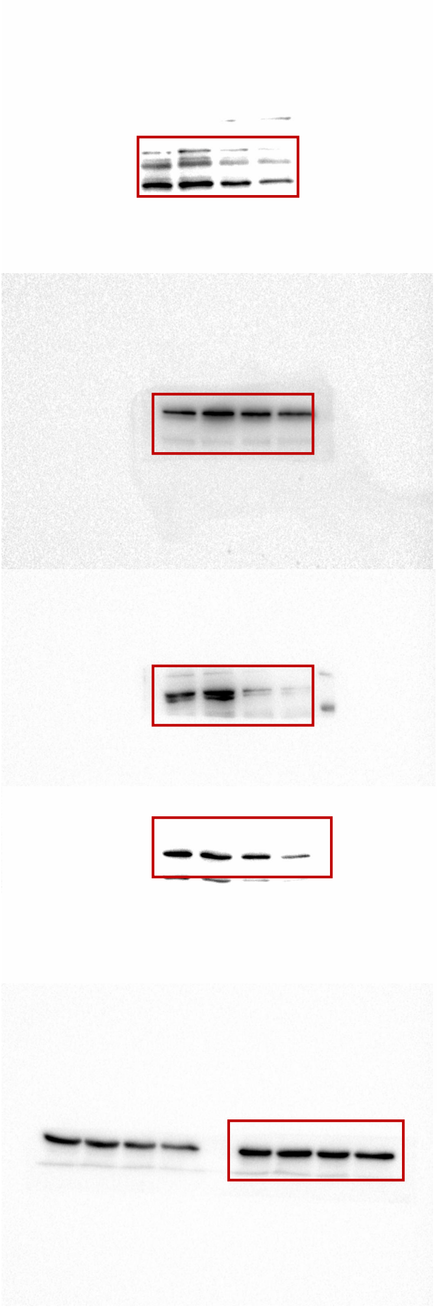

Figure 4

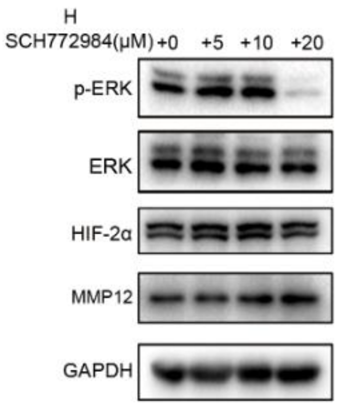
H
SCH772984(μM) +0 +5 +10 +20
p-ERK
ERK
HIF-2α
MMP12
GAPDH

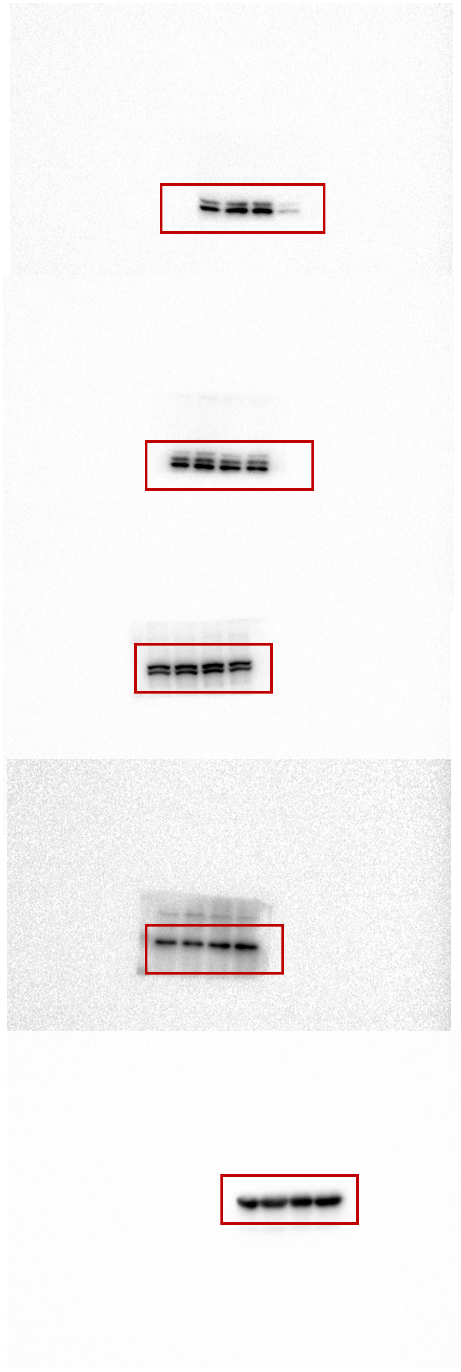

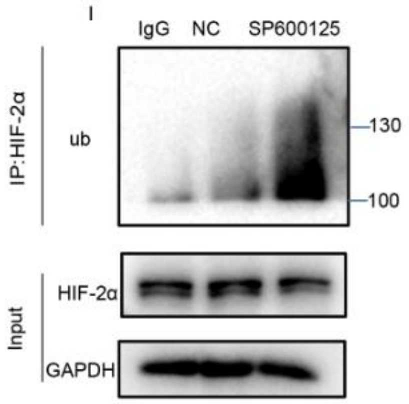
I
IgG NC SP600125
IP:HIF-2α
ub
130
100
Input
HIF-2α
GAPDH

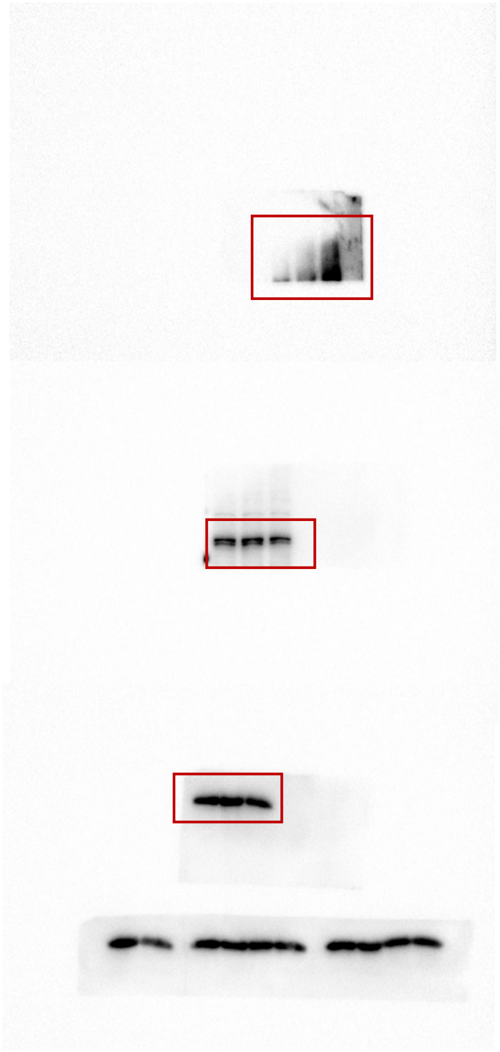

Figure 5

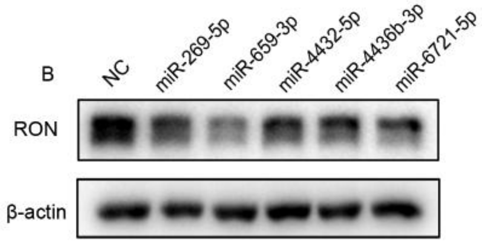
B
NC
miR-269-5p
miR-659-3p
miR-4432-5p
miR-4436b-3p
miR-6721-5p
RON
β-actin

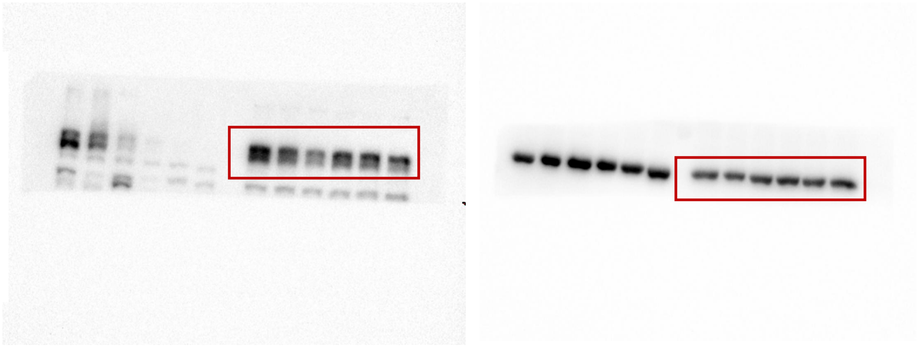

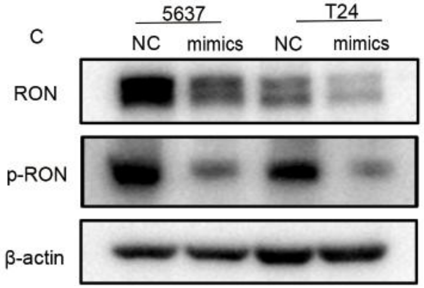
C
5637
T24
NC
mimics
NC
mimics
RON
p-RON
β-actin

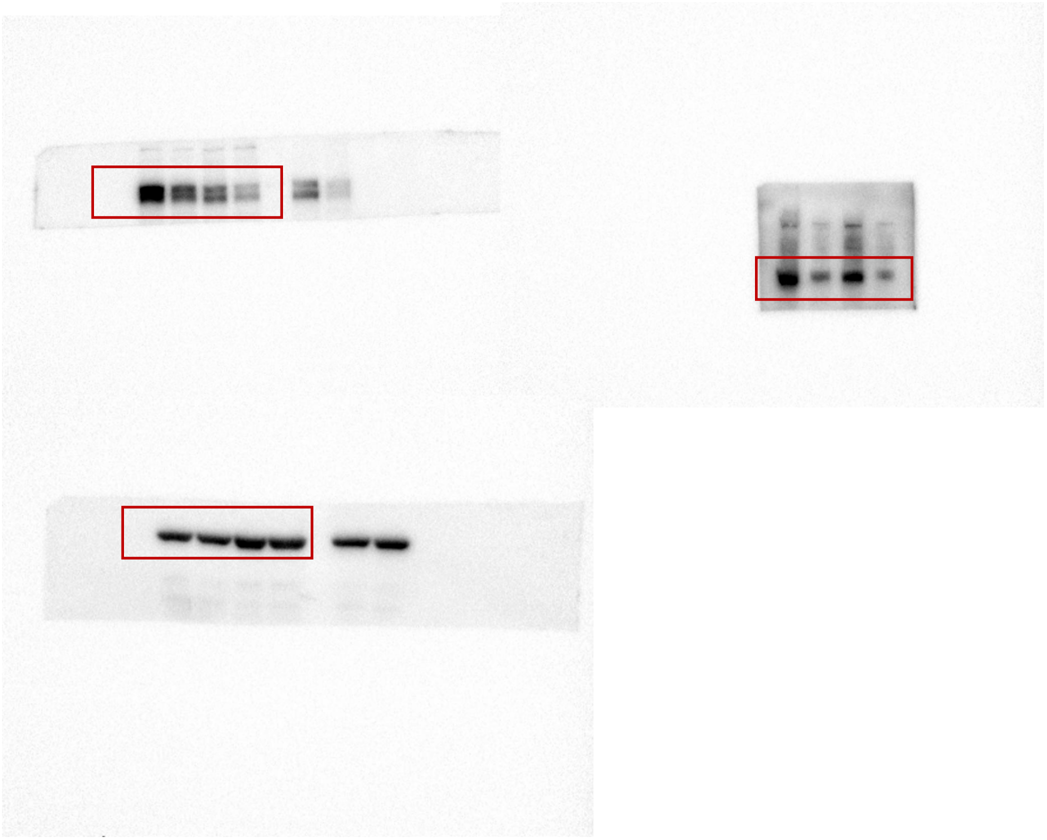

Figure 6

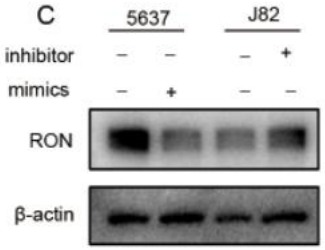
C
5637
J82
inhibitor – – – +
mimics – + – –
RON
β-actin

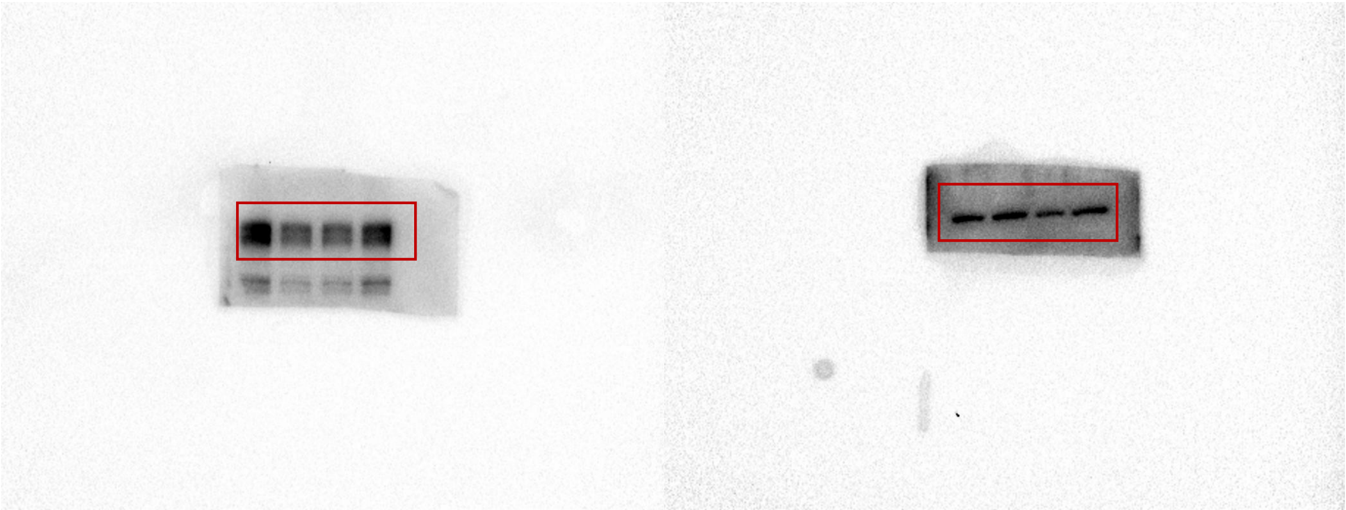

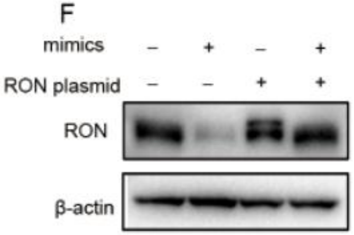
F
mimics – + – +
RON plasmid – – + +
RON
β-actin

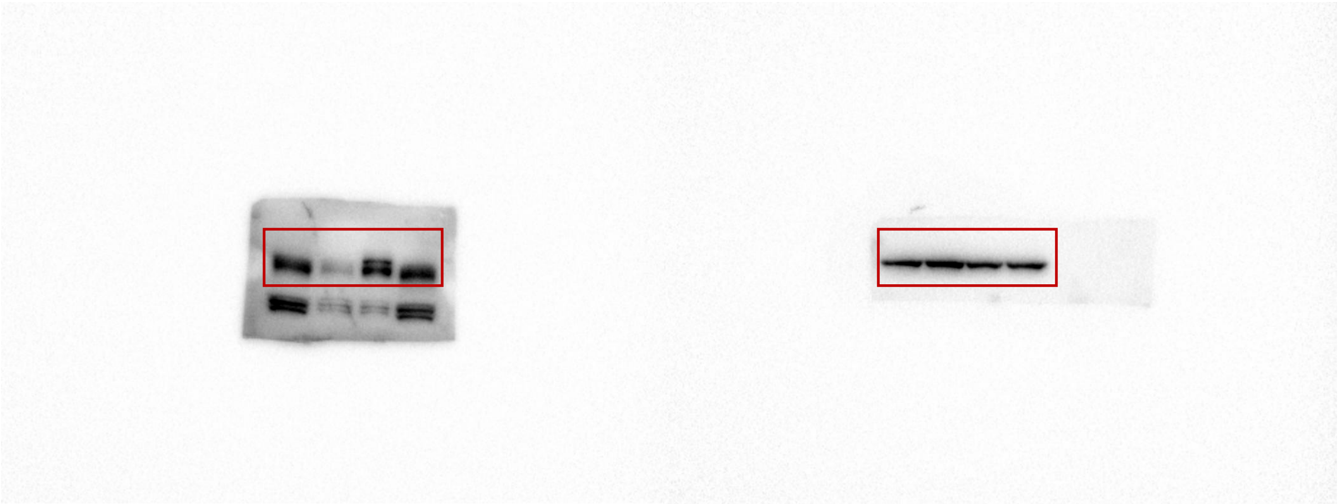

Supplementary Figure 1

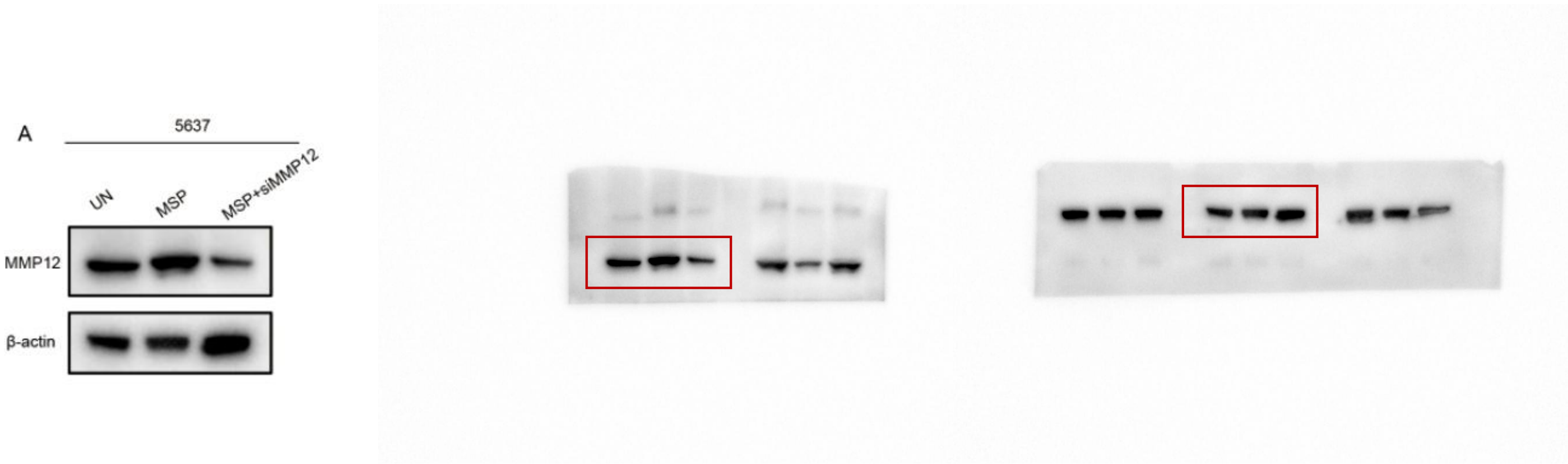

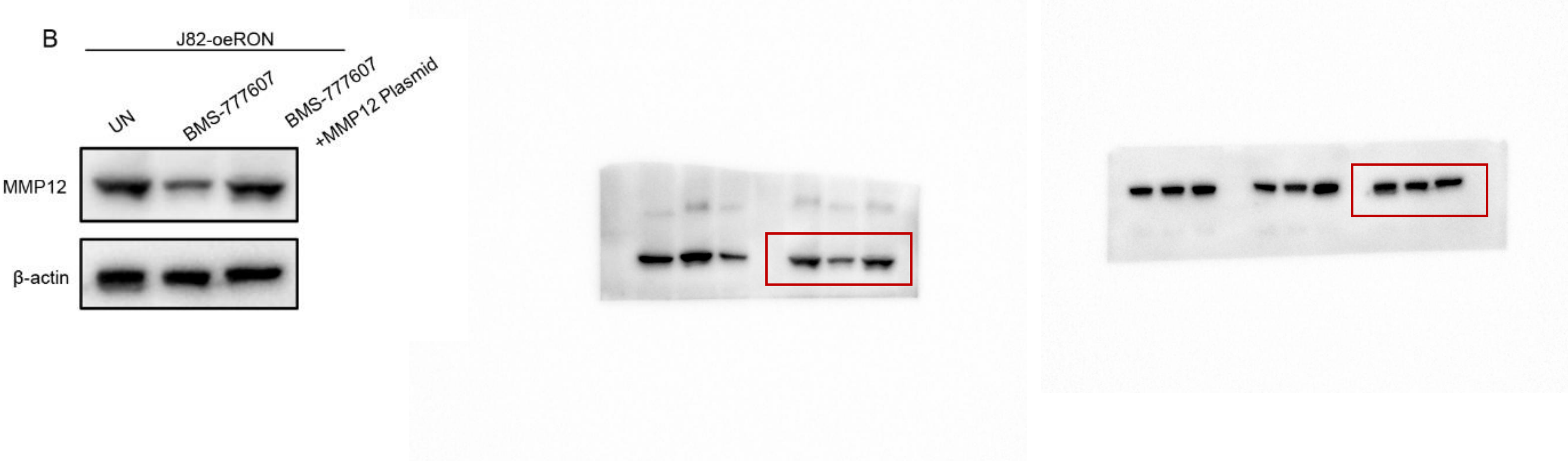

Supplementary Figure 2

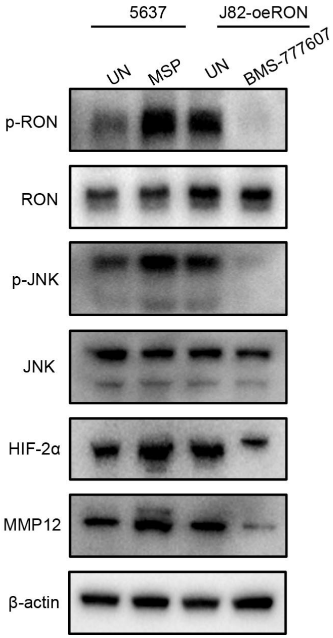

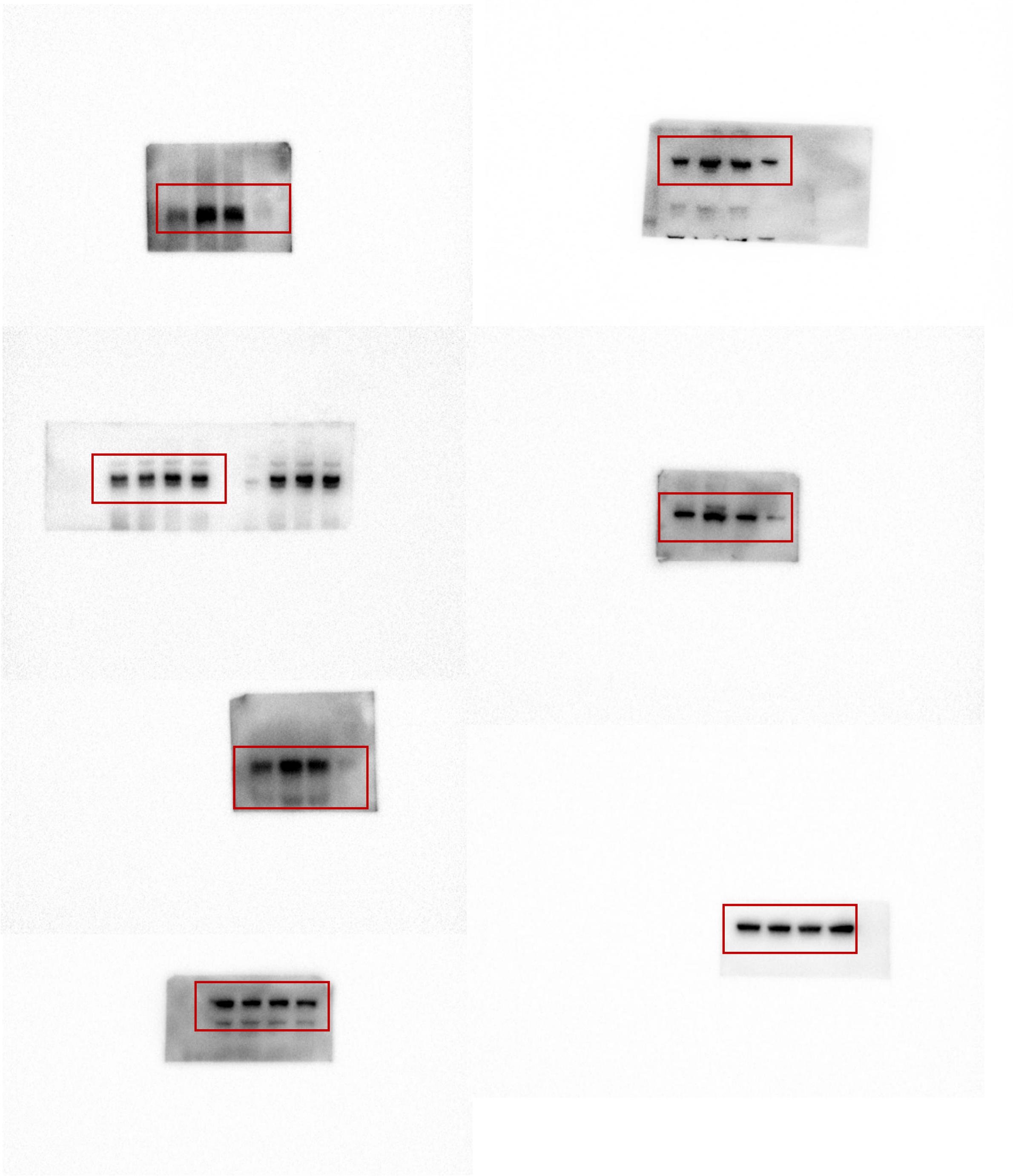

Supplementary Figure 3

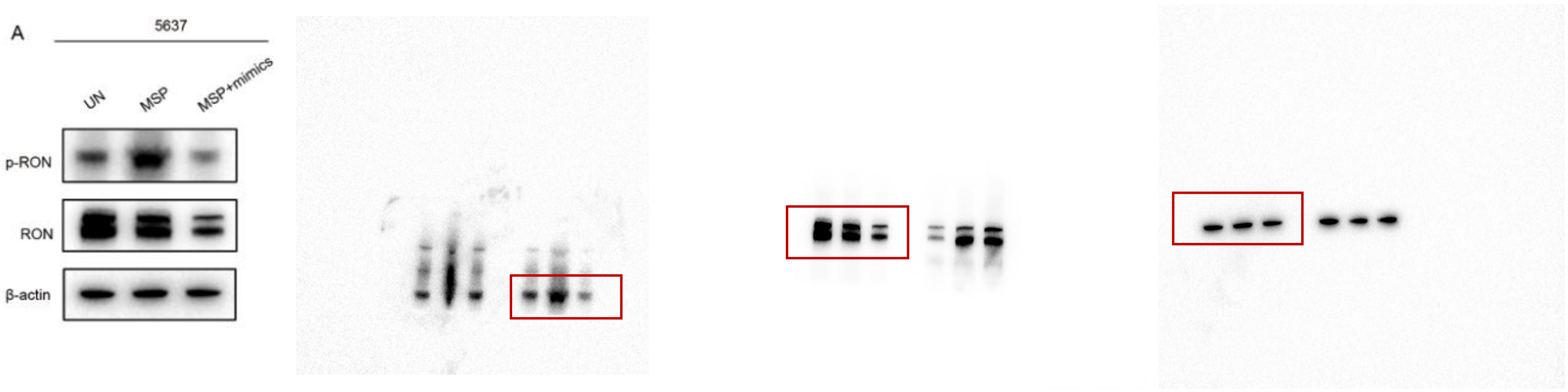

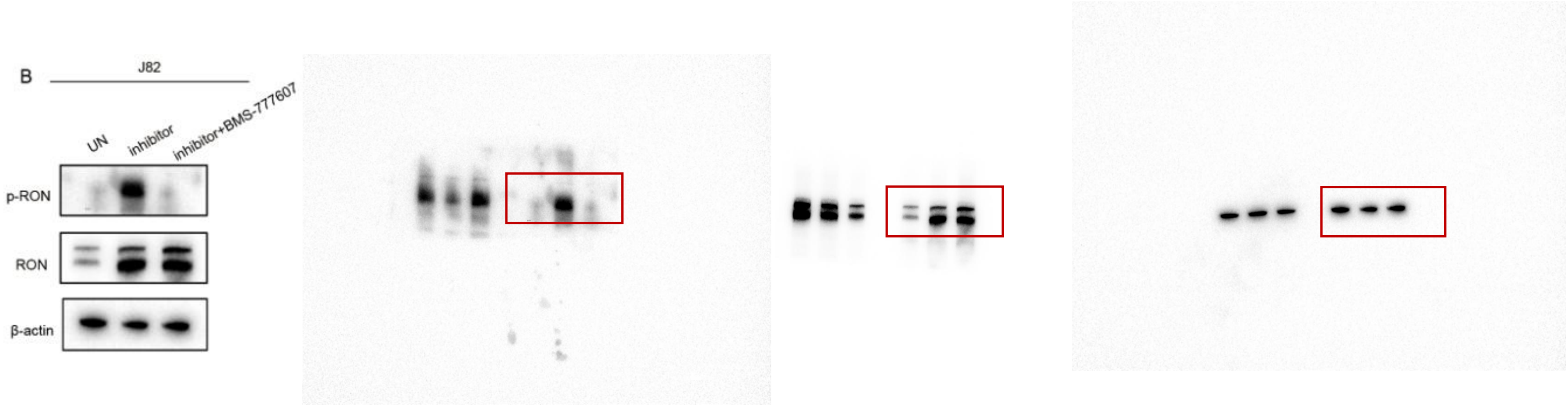

Supplement: Supplementary file 3 — wb original data [file 41419_2024_7245_MOESM3_ESM.pdf]
